# Targeting Human Immunodeficiency Virus Integrase Beyond the Active Site: The Discovery and Development of Allosteric Integrase Inhibitors

**DOI:** 10.1002/cmdc.70428

**Published:** 2026-08-22

**Authors:** Francesco Saccoliti, Elisa Patacchini, Emanuele Cara, Laura Zarbo, Antonella Messore, Aurora Albano, Giuseppe Ruggieri, Valentina Noemi Madia, Luigi Scipione, Roberta Costi, Roberto Di Santo

**Affiliations:** ^1^ Department of Life Science Health and Health Professions Link Campus University Rome Italy; ^2^ Dipartimento di Chimica e Tecnologie del Farmaco Istituto Pasteur‐Fondazione Cenci Bolognetti “Sapienza” Università di Roma Rome Italy; ^3^ Dottorato di Interesse Nazionale in One Health Approaches to Infectious Diseases and Life Science Research Dipartimento di Sanità Pubblica Medicina Sperimentale e Forense Università degli Studi di Pavia Pavia Italy; ^4^ Department of Science Roma Tre University Rome Italy

**Keywords:** allosteric integrase inhibitor, antiviral agents, drug discovery, integrase, structure–activity relationships

## Abstract

Given its pivotal role in the viral life cycle, blocking integrase (IN) through IN strand transfer inhibitors (INSTIs) represented a breakthrough in the treatment of HIV infection, establishing these antiretroviral regimens as first‐line options against AIDS. However, the onset of drug‐resistant strains has challenged the efficacy of INSTIs, demanding new efforts in the search for therapeutic tools that work through alternative modes of action. In this context, the discovery of allosteric IN inhibitors (ALLINIs) has highlighted new opportunities to target IN beyond its active site, enhancing antiviral efficacy and the genetic barrier to resistance. Extensive drug discovery campaigns have resulted in the development of effective ALLINIs with both in vitro and in vivo efficacy, two of which are advancing in clinical trials as next‐generation therapeutic tools. Nevertheless, advancements in structural biology have critically aided in elucidating the underlying effects of ALLINIs, highlighting a complex, multimodal mode of action that ultimately yields defects in virion maturation. This review focuses on ALLINIs, providing a comprehensive overview of major drug discovery efforts devoted to this field, with an emphasis on the medicinal chemistry and structural biology findings that have revealed unprecedented opportunities for developing innovative and effective anti‐HIV drugs.

## Graphical abstract

Human immunodeficiency virus (HIV) allosteric integrase inhibitors (ALLINIs) offer a vital alternative to standard therapies by targeting noncatalytic sites. By inducing aberrant integrase multimerization, they disrupt viral maturation. This review explores their medicinal chemistry evolution, detailing structural insights, structure–activity relationships (SAR), and the promising clinical candidates currently in development.

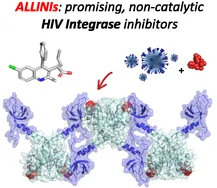

## 1 Introduction

The human immunodeficiency virus type‐1 (HIV‐1) is a retrovirus that progressively attacks and weakens the immune system, specifically targeting CD4^+^T cells. If left untreated, HIV infection leads to acquired immunodeficiency syndrome (AIDS), the clinical stage defined by profound immunosuppression and susceptibility to opportunistic infections [1]. Since the start of the epidemic, an estimated 91.4 million people have acquired HIV, with approximately 40.8 million people living with the virus globally by the end of 2024 [2]. Despite these, significant advances in the antiretroviral therapy (ART) have transitioned AIDS from a fatal diagnosis to a manageable chronic condition, significantly extending the lifespan and improving the quality of life of affected patients [3].

In the search for effective antiretroviral agents, targeting the viral enzyme integrase (IN) has emerged as a cornerstone of modern ART, offering a vital alternative for patients who have developed resistance to traditional ART regimens. The discovery of drugs targeting a specific step of the integration process, referred to as IN strand transfer inhibitors (INSTIs), has marked a key breakthrough in this area. Subsequent optimization of first‐generation inhibitors led to the development of second‐generation INSTIs, which offer superior genetic barriers to resistance and enhanced pharmacokinetic (PK) profiles, establishing them as frontline components of modern ART [4, 5, 6]. However, despite these great achievements, HIV treatment remains a substantial burden. The virus's exceptionally high mutation rate frequently renders established therapeutic regimens ineffective over time. This concern is further underscored by recent findings that second‐generation INSTIs can select for resistant mutants [7, 8, 9, 10]. Some of these variants exhibit cross‐resistance to other antiretrovirals, including INSTIs, highlighting the impelling need to explore alternative mechanisms to inhibit HIV‐1 effectively.

Over the last two decades, targeting IN beyond its active site through noncatalytic, allosteric IN inhibitors (ALLINIs) has emerged as a promising, complementary strategy to enhance antiviral potency and limit the selection of mutant strains. In this scenario, disrupting the interaction between IN and the host cofactor lens epithelium–derived growth factor (LEDGF/p75) led to the development of early protein–protein interaction ALLINIs with significant antiviral effects and notable genetic barriers to resistance. Notably, they showed no cross‐resistance with existing antiretrovirals, making them ideal candidates for supplementing current ART regimens. In addition to elucidating the underlying basis for ALLINI activity, insightful studies have aided in unveiling previously unrecognized aspects of HIV‐1 biology, opening new avenues for therapeutic intervention and highlighting new opportunities to target IN outside its catalytic site [11, 12, 13]. Continuous research has since identified several promising ALLINIs with both in vitro and in vivo efficacy, some of which have advanced in clinical trials as next‐generation therapeutic options.

This review provides an extensive overview of ALLINIs. Following a stepwise historical perspective, we discuss the discovery and development of this class of inhibitors, highlighting both the key insights clarifying their unique mechanism of action, and the medicinal chemistry efforts which have ultimately led to some of the most promising and advanced anti‐HIV‐1 candidates. Furthermore, we highlight a set of recent inhibitors which, despite sharing similarities with ALLINIs, demonstrate a distinct dual‐mode of inhibition by targeting both the catalytic activity of IN and IN–RNA binding.

### 1.1 HIV‐1 Life Cycle

HIV‐1 belongs to the *Retroviridae* family and is an enveloped virus characterized by an icosahedral capsid enclosing the viral genome. As with all retroviruses, the genome consists of two copies of a positive sense single‐stranded RNA (ssRNA) [14]. HIV‐1 selectively packages the viral genome and introduces specific modifications to the +ssRNA [15, 16]. The viral genome encodes three structural polyproteins (Gag, Pol, and Env) as well as six accessory proteins (Tat, Rev, Nef, Vpr, Vif, and Vpu), all of which play essential roles in viral replication and pathogenesis. HIV‐1 entry into host cells is mediated by the viral envelope glycoproteins gp120 and gp41, which recognize the CD4 receptor in combination with the CCR5 or CXCR4 coreceptors, expressed on target cells such as T helper lymphocytes and microglial cells [15, 16, 17]. Following receptor engagement and membrane fusion, the viral core is released into the cytoplasm, where the viral replication cycle begins.

A hallmark of HIV‐1 replication is the reverse transcription of the ssRNA genome into double‐stranded DNA (dsDNA). Reverse transcription is carried out by the virally encoded reverse transcriptase (RT), a multifunctional enzyme that exhibits both RNA‐dependent DNA polymerase activity, responsible for the synthesis of the RNA:DNA intermediate, and ribonuclease H (RNase H) activity, which selectively degrades the RNA strand of the RNA:DNA hybrid [18, 19]. Following reverse transcription, the newly synthesized viral dsDNA is transported to the nucleus, where another viral enzyme, IN, catalyzes its insertion into the host genome through a tightly regulated integration process. Finally, as observed for other retroviruses, viral maturation requires the activity of the HIV‐1 protease (PR), which cleaves the Gag and Gag‐Pol polyproteins into functional viral components, yielding infectious virions.

### 1.2 HIV‐1 IN

A defining hallmark of retroviral replication is the obligatory integration of the viral genome into the host DNA, a feature that clearly distinguishes retroviruses from all other viral families. This critical step is mediated by the viral enzyme IN, which, together with RT and PR, constitutes the canonical enzymatic triad encoded by replication‐competent retroviruses [11, 12, 20].

HIV‐1 IN is a member of the polynucleotidyl transferase superfamily and is a 32 kDa protein composed of 288 amino acids, functioning as a higher‐order multimer and organized into three distinct structural domains [12]. The N‐terminal domain (NTD) contains a conserved zinc‐binding motif that coordinates a Zn^2+^ ion, ensuring proper protein folding and multimerization. The catalytic core domain (CCD) harbors the conserved DDE catalytic triad (D64, D116, and E152), which coordinates two Mg^2+^ ions essential for IN catalytic activity. Finally, the C‐terminal domain (CTD) is involved in specific interactions with both viral and host DNA.

**FIGURE 1:**
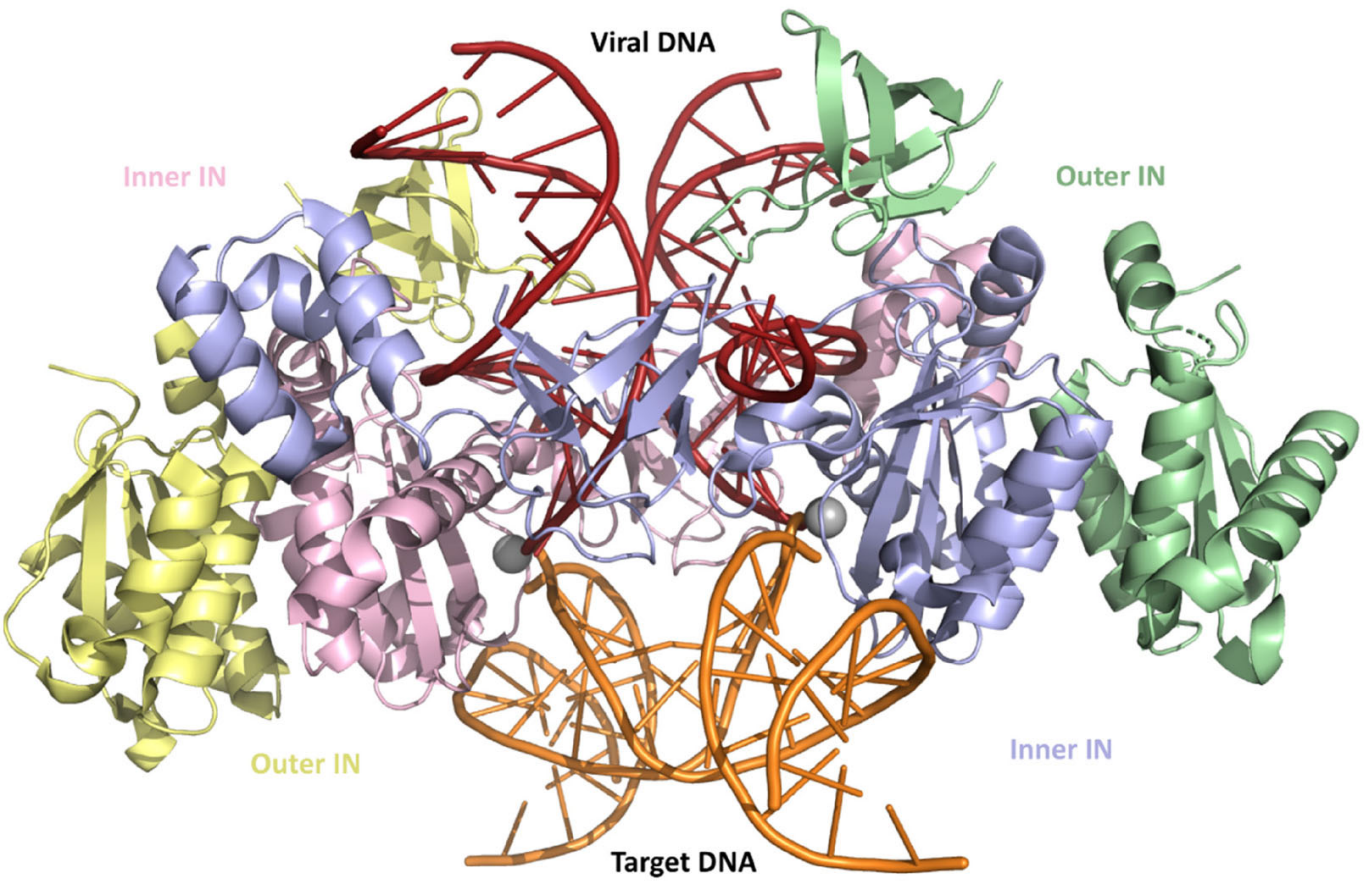
Structure of tetrameric HIV‐1 strand transfer complex intasome. Cartoon representation of the HIV‐1 stable synaptic complex after strand transfer reaction, as determined by cryoEM (PDB: 5U1C). The viral DNA is presented as ladders in red, whereas the target DNA is colored in orange. The IN protomers bound to DNA (inner INs) are shown as ribbons in light blue and pink, the other IN protomers (outer INs) are rendered as ribbons in light green and yellow. Magnesium ions are shown as gray spheres.

HIV‐1 IN functions as a multimer, and all three domains contribute to the formation and stabilization of its functional oligomeric state (Figure 1) [12, 18, 21, 22].

To gain detailed insights into the mechanism of HIV‐1 DNA integration, prominent efforts have been devoted to obtaining high‐resolution structures of key integration intermediate nucleoprotein complexes. However, structural studies of HIV‐1 intasomes have proven challenging, largely due to the propensity of HIV‐1 IN and the assembled complexes to undergo aggregation [21, 22, 23].

While early studies on intasomes relied on related retroviruses (such as prototype foamy virus, PFV) [24, 25, 26, 27] and homology modeling for HIV‐1 [28, 29], advances in cryo‐EM have ultimately enabled the determination of high‐resolution structures [22]. These revealed a dynamic organization containing a minimum of four IN subunits and as many as 16 subunits, all arranged around the central viral DNA ends. Notably, this has marked a key milestone in the field, highlighting unprecedented features of the unique architecture of the HIV‐1 intasome (Figure 1) [11, 12, 22, 30].

In addition, this achievement has catalyzed the resolution of related structures encompassing bound INSTIs, yielding key insights into their underlying mechanism of action [31]. Notably, these structures revealed key differences in drug binding that were previously unappreciated in studies using PFV intasome surrogates. By revealing in‐depth details at near‐atomic resolution, these findings have helped to conclusively rationalize how such inhibitors bind to their native target, providing key insights for rationally designing next‐generation inhibitors while explaining the molecular basis for drug‐resistant HIV‐1 phenotypes [4, 11, 12, 31, 32, 33].

**FIGURE 2:**
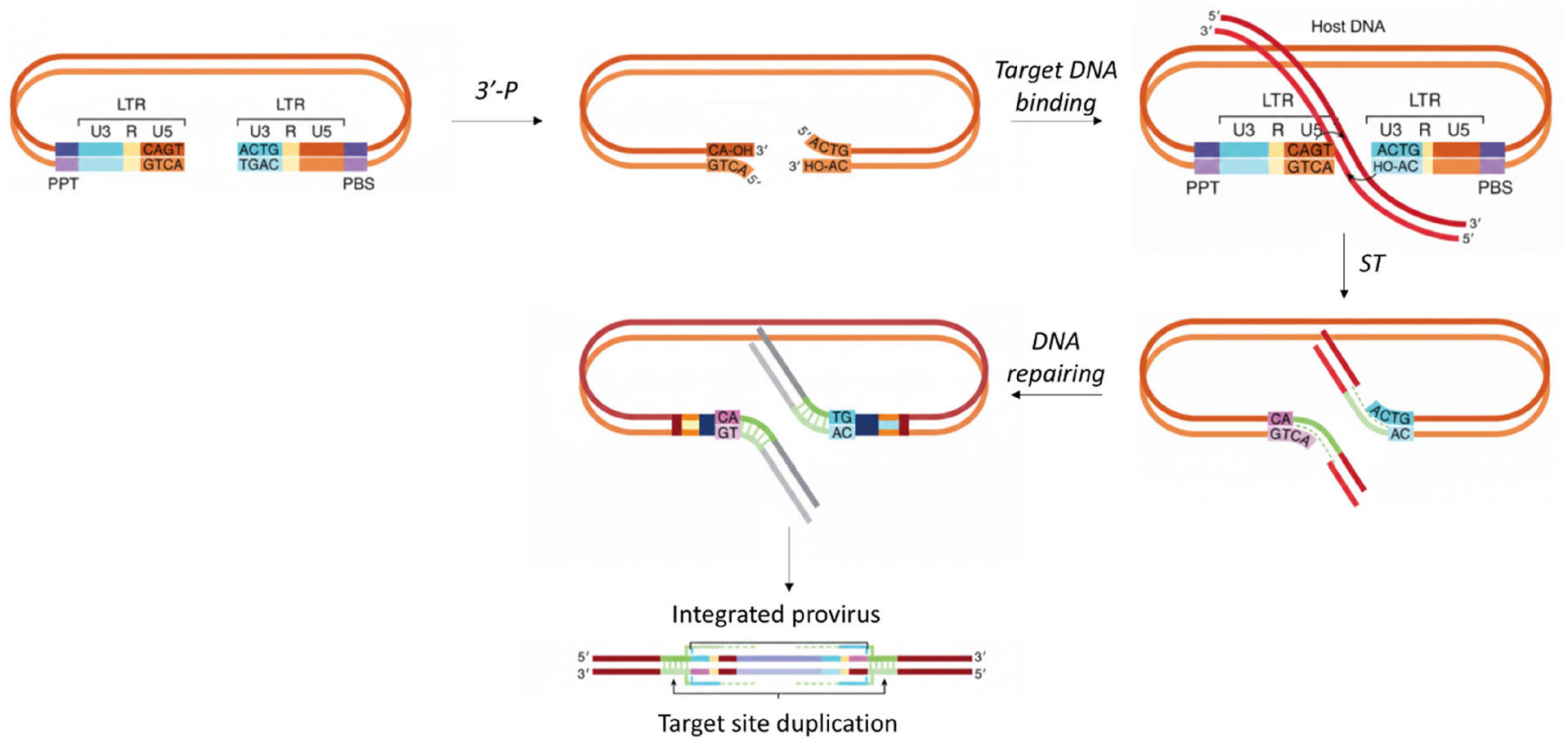
The linear viral reverse transcript (orange lines; plus‐strands shaded more darkly than the yellow minus‐strands throughout the diagram) contains a copy of the LTR at each end. These LTRs are composed of blue U3, yellow R repeat, and light‐orange U5 sequences. The upstream LTR is bordered by the primer‐binding site (PBS), while the downstream element is adjacent to the polypurine tract (PPT). During the 3′‐P, IN hydrolyzes the DNA strands immediately next to the invariant CA dinucleotides. In the case of HIV‐1, this reaction liberates the pGT‐OH dinucleotide from the ends of the viral DNA. Following nuclear translocation, the intasome engages with the host target DNA (represented by red lines) to mediate DNA strand transfer. The remaining DNA gaps are subsequently repaired by the host cell machinery, yielding a target site duplication (thin green lines) that flanks the integrated provirus.

HIV‐1 IN mediates the insertion of the retrotranscribed viral dsDNA into the host cell genome through two sequential and spatially distinct catalytic steps. In its multimeric form, the enzyme assembles a stable synaptic complex (SSC) with viral DNA [12, 22]. The first reaction occurs in the cytoplasm within the preintegration complex (PIC), a nucleoprotein assembly that includes viral enzymes and host factors to shield the viral genome from degradation. This step, termed 3′‐processing (3′‐P), involves the removal of a GT dinucleotide from each 3′‐end of the viral long terminal repeats (LTRs). The second reaction, known as strand transfer (ST), takes place in the nucleus and results in the covalent integration of the processed viral DNA into host chromosomal DNA, followed by gap repair mediated by the host DNA repair machinery (Figure 2) [11, 12, 20, 30, 34].

**FIGURE 3:**
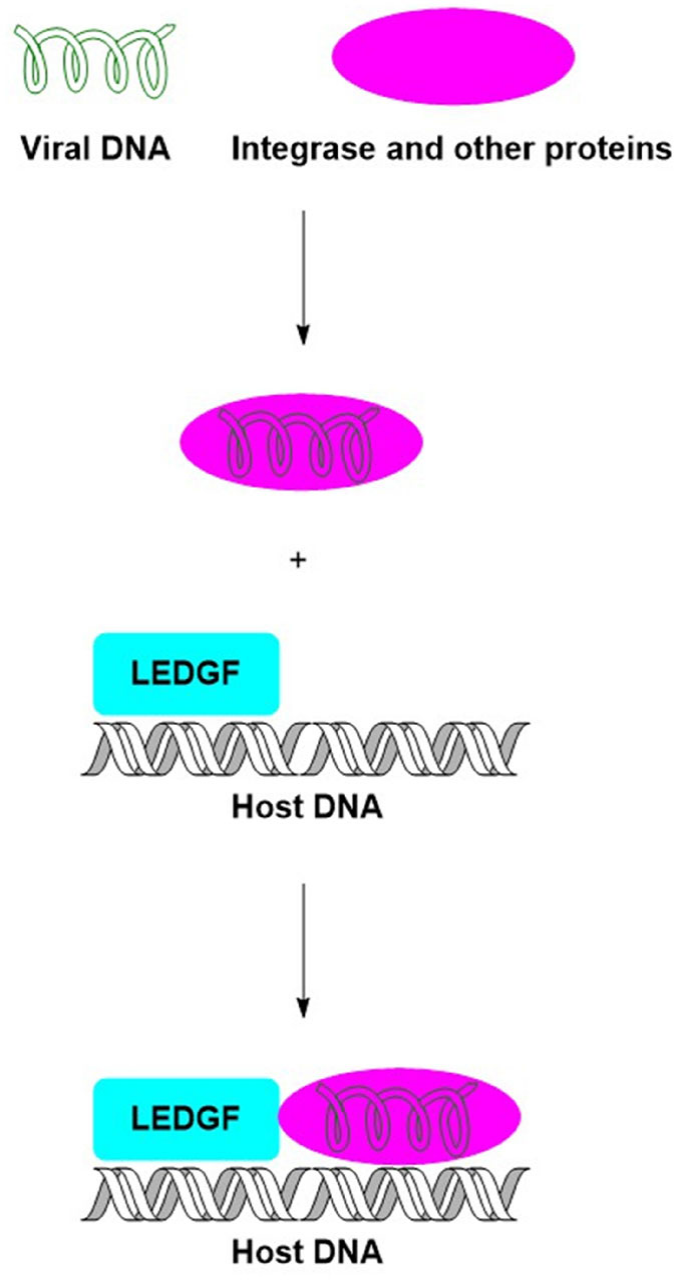
LEDGF/p75 recruits the HIV PIC and promotes HIV integration at a nearby site. The PIC, depicted in purple, is composed of IN and additional viral/host proteins associated with the viral DNA. Viral IN interacts with LEDGF/p75 (shown in light blue), forming a stable complex.

Among the cellular cofactors involved in facilitating the integration process, LEDGF/p75 [35, 36] plays a key role by tethering IN to chromatin through specific interactions between its IN binding domain (IBD) and the V‐shaped pocket at the CCD dimer interface [37, 38]. LEDGF/p75 is a nuclear protein belonging to the hepatoma‐derived growth factor (HDGF)‐related protein (HRP) family [39]. A defining feature of this family is the presence of a highly conserved N‐terminal PWWP domain, which is involved in chromatin binding and protein–protein interactions. In the LEDGF/HRP family, this specifically appears as a modified proline–histidine–tryptophan–proline (PHWP) motif. Its key functional role, however, resides in the C‐terminal region, which contains the IBD. This domain enables direct interaction with HIV‐1 IN, acting as a molecular tether that anchors the PIC to host chromatin and facilitates its targeting to transcriptionally active regions, thereby directing viral DNA integration across active gene bodies (Figure 3). The interaction is mediated by approximately 80 residues within the IBD, which primarily engage the CCD of IN, with additional contributions from the NTD in enhancing binding affinity. Furthermore, LEDGF/p75 stabilizes IN by shielding it from proteasomal degradation, thereby promoting efficient viral integration and productive replication [36, 40, 41, 42, 43].

### 1.3 HIV‐1 INSTIs

Owing to its essential function in the viral replication cycle and its well‐established druggability, HIV‐1 IN has become a major target for antiretroviral drug discovery. Since the emergence of HIV infection, extensive efforts have led to the development of numerous IN inhibitors, several of which are now routinely employed in therapy. All IN inhibitors currently approved for clinical use belong to the class of INSTIs, which selectively block the ST reaction by chelating the two Mg^2+^ ions within the CCD, thereby preventing the stable integration of the viral dsDNA into the host genome [4, 5, 6, 12, 32, 44, 45]. The discovery of diketoacid (DKA) derivatives as selective INSTIs represented an important milestone in the field, providing early evidence for validating IN as a viable pharmacological target. These compounds feature a DKA pharmacophore consisting of a coplanar, metal‐coordinating oxygen triad linked to a heteroaromatic core and a halobenzyl moiety. The latter occupies a narrow pocket (normally reserved for the 3′‐terminal base of the viral DNA) created by the ejection of the invariant GT dinucleotide after the 3′‐P. Here, the halobenzyl moiety interacts with the penultimate cytidine at the 3′ end of the DNA reactive strand, displacing the terminal 3′‐adenosine through an induced‐fit mechanism [5, 24, 28, 45, 46, 47, 48, 49, 50, 51].

**FIGURE 4:**
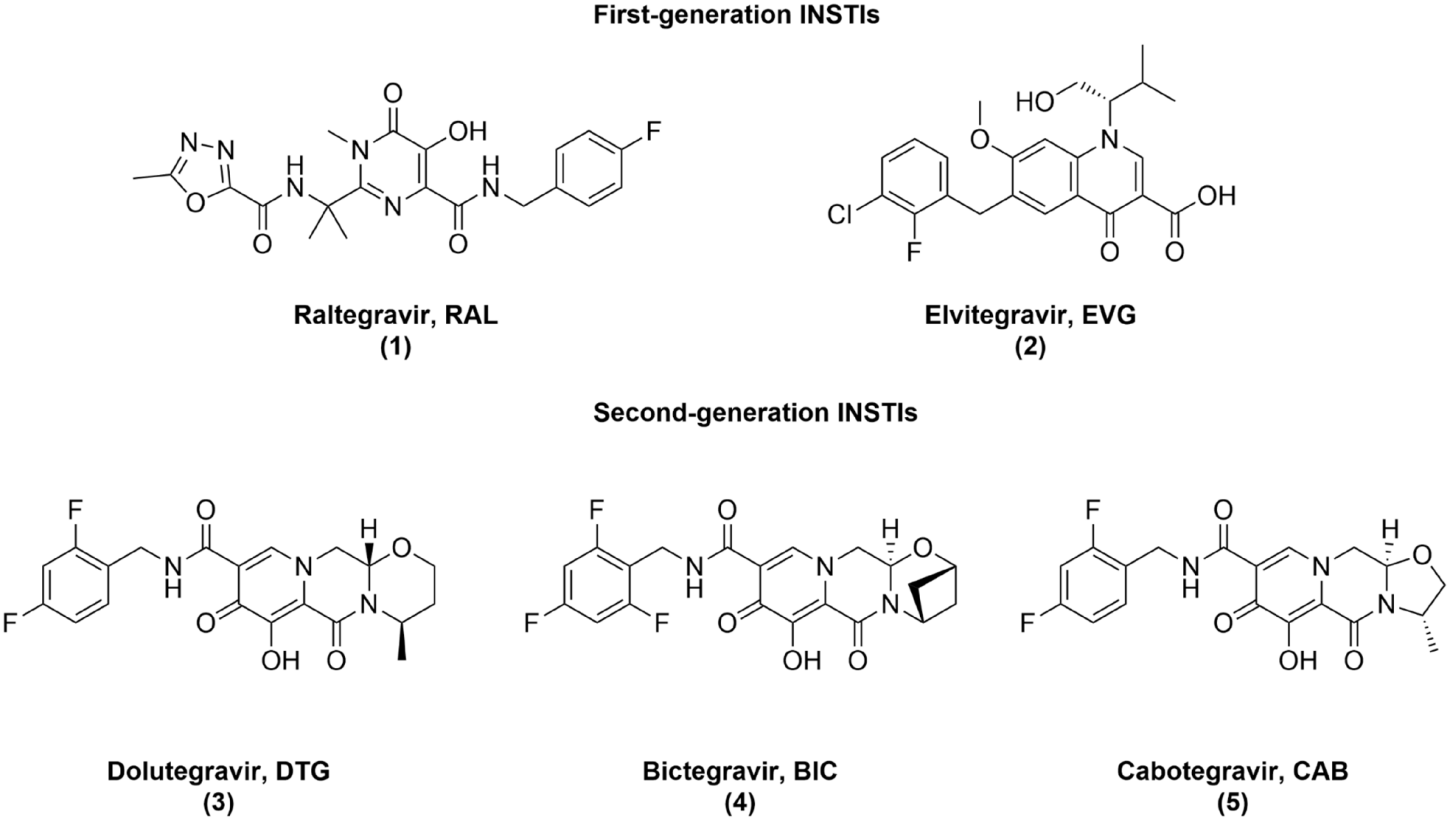
Chemical structures of INSTIs. First‐generation INSTIs: RAL (**1**) and EVG (**2**) and second‐generation INSTIs: DTG (**3**), BIC (**4**) and CAB (**5**).

Optimization efforts focused on the modification of the DKA scaffold to lock the pharmacophore into a coplanar conformation in DKA bioisosteric analogs. These structural refinements led to the development of potent in vitro and in vivo inhibitors, eventually resulting in the first approved INSTIs raltegravir (RAL, **1**, Figure 4) and elvitegravir (EVG, **2**, Figure 4) [4, 5, 45, 48, 52, 53, 54, 55].

Although the discovery of RAL and EVG marked a major milestone in ART, their clinical utility was frequently hampered by suboptimal PK profiles and a low genetic barrier to resistance. Specifically, the rapid development of single or combined IN mutations often led to relevant cross‐resistance, compromising the extensive use of these first‐generation INSTIs [4, 5, 30, 33, 45, 56, 57, 58].

To address these limitations, prominent medicinal chemistry efforts were undertaken to identify effective INSTIs with improved antiviral efficacy, a higher genetic barrier to resistance, and favorable PK properties. These investigations focused on the identification of inhibitors bearing a more conformationally constrained metal‐binding pharmacophore, exemplified by the tricyclic carbamoyl‐pyridone scaffold of the second‐generation INSTI dolutegravir (DTG, **3**, Figure 4). Of note, this scaffold retains the essential triad of heteroatoms required for Mg^2+^ chelation while improving geometric preorganization and reducing entropic penalties upon binding. Subsequent optimizations focused on fine‐tuning interactions within the IN–viral DNA complex through the introduction of optimized hydrophobic substituents capable of occupying adjacent pockets and reinforcing π–π stacking with viral DNA bases. These efforts led to the development of structurally related analogus such as bictegravir (BIC, **4**, Figure 4) and cabotegravir (CAB, **5**, Figure 4), which share the same metal‐chelating scaffold as DTG but incorporate additional structural refinements, including expanded bicyclic systems and tailored side chains [4, 5, 12, 30, 33, 44, 57, 58, 59, 60, 61, 62, 63, 64].

Such modifications have significantly broadened the efficacy of INSTIs against challenging viral strains resistant to first‐generation INSTIs, while also providing slower dissociation kinetics from the intasome [5, 30, 64, 65, 66]. Importantly, the improved PK characteristics of second‐generation INSTIs have translated into significant clinical advantages, including once‐daily unboosted regimens and, in the case of CAB, long‐acting injectable formulations administered monthly or at even longer intervals. This reduced dosing frequency markedly improves treatment adherence and represents a major advancement not only for therapeutic regimens but also for preventive strategies. Notably, long‐acting CAB has been approved for pre‐exposure prophylaxis (PrEP), underscoring the transformative impact of second‐generation INSTIs beyond treatment and into HIV prevention [4, 5, 6, 63, 64, 65, 66, 67, 68, 69, 70].

Noteworthy, the successful optimization of these second‐generation agents was crucially informed by the extensive structure–activity relationship (SAR) and resistance data gathered from first‐generation INSTIs, alongside advancements in the structural and functional understanding of IN [4, 5, 11, 12, 24, 28, 30, 31, 32, 33, 44, 60, 61, 62, 64].

Despite these, the emergence of viral strains with reduced susceptibility to INSTIs remains a critical challenge [7, 8, 9, 10, 71, 72, 73]. Beyond the primary mutations identified under selective pressure from second‐generation INSTIs [5, 12, 71, 72, 73, 74, 75], significant concern has shifted toward distal substitutions located away from the active site. Structural studies have indicated that these residues are not directly involved in INSTI binding; instead, they contribute to the secondary coordination sphere of the catalytic Mg^2+^. Mutations at these sites can perturb the precise coordination geometry of the metal ions, thereby indirectly modifying the electronic and spatial environment of the catalytic core and resulting in impaired inhibitor affinity [4, 12, 32, 33].

## 2 HIV‐1 ALLINIs

**FIGURE 5:**
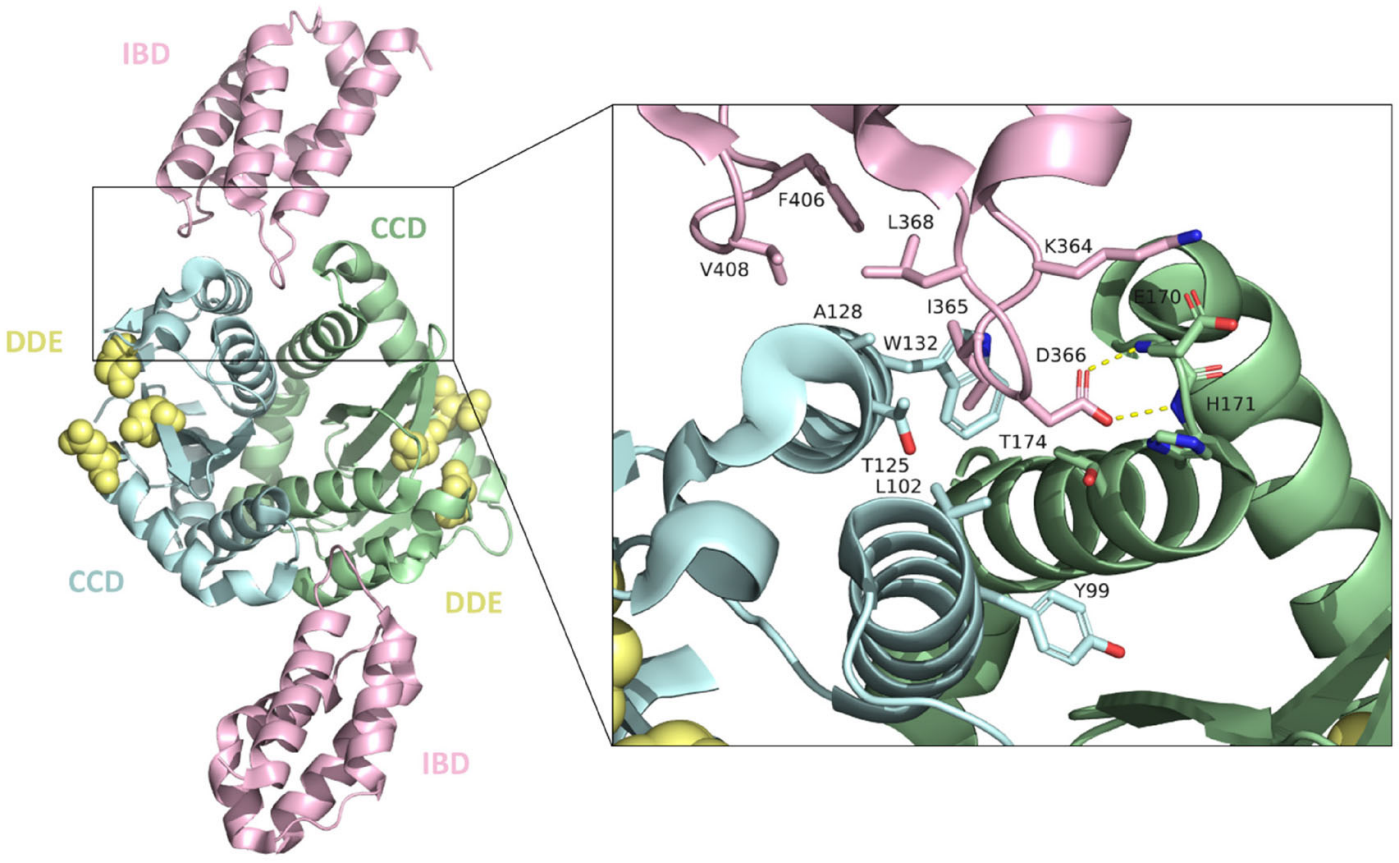
Crystal structure of dimeric CCD of HIV‐1 IN in complex with LEDGF/p75 IBD (PDB: 2B4J). Overall view of the CCD dimer of IN in complex with LEDGF/p75 IBD. On the right panel, a close view focused on CCD–IBD binding. The proteins are shown as cartoons, with IN CCD dimer chains colored in light green and light cyan and the LEDGF/p75 IBDs in light pink, while the catalytic triad residues are shown as yellow spheres.

Although the discovery of INSTIs has profoundly transformed HIV therapy by expanding the therapeutic options for the treatment of AIDS, the continued emergence of drug‐resistant viral strains has represented a major clinical challenge. Resistance to both first‐ and second‐generation INSTIs has been reported, particularly in treatment‐experienced patients with multidrug‐resistant HIV strains [7, 8, 9, 10, 12, 76, 77]. These observations have spurred the development of new antiretroviral agents targeting unexplored steps of viral life cycle to overcome drug resistance. Accordingly, since the approval of RAL, considerable attention has been devoted to the identification of allosteric inhibitors of IN, with a primary focus on disrupting the interaction between IN and host factors [30, 42, 78]. In this context, most research has focused on targeting the interaction between HIV‐1 IN and the cellular cofactor LEDGF/p75 [79, 80, 81]. LEDGF/p75 binds IN through its C‐terminal IBD, which engages a V‐shaped pocket at the CCD–CCD dimer interface, a region distinct from the catalytic site. Crystallographic studies have revealed that this interface forms a well‐defined binding pocket stabilized by hydrophobic interactions and hydrogen bonds [38, 82]. Mutagenesis experiments have further confirmed the critical role of residues from both IN and LEDGF/p75 in mediating this interaction (Figure 5) [38, 45, 83, 84, 85, 86, 87]. Specifically, LEDGF/p75 interacts with E170 and H171 of one subunit of IN via bidentate H‐bonds mediated by D366, while the IBD residue I365 nestles into a hydrophobic pocket composed by L102, A128, and W132 from the other IN subunit (Figure 5). First‐generation ALLINIs were indeed pharmacologically designed to recapitulate these specific polar and nonpolar interactions, effectively mimicking the binding signatures of the natural cofactor [30, 38, 42, 45, 78, 79, 84, 88].

Notably, several of these interface residues are central to the binding affinity and clinical profile of ALLINIs. Specifically, all ALLINIs retain a characteristic carboxylic group to mimic the bidentate interaction between LEDGF/p75 D366 and IN, alongside an aromatic side chain that occupies the W132/L102 cleft and recapitulates the hydrophobic role of I365 [20, 30, 42].

Among these residues, T125 is of clinical relevance as it is subject to natural polymorphism; the prevalence of alanine at this position (A125) in several viral clades can inherently reduce the baseline susceptibility to certain inhibitor scaffolds [89, 90].

Additionally, A128 plays a pivotal role in the emergence of viral resistance. Indeed, being frequently targeted by mutations, the replacement of this alanine with a bulkier or polar residue, such as threonine (A128T), introduces steric constraints that hinder inhibitor binding [30, 91].

Similarly, residue Y99 is often subject to mutations under drug pressure, usually compromising the stability of the inhibitors within the allosteric pocket [92].

Based on these structural insights, disruption of the LEDGF/p75–IN interface by small molecules emerged as a feasible strategy, particularly amenable to structure‐based drug design approaches owing to the availability of high‐resolution structural data [38, 42, 45, 82, 93].

The mechanism of action of ALLINIs has been extensively investigated and elucidated over the years. Strikingly, while early efforts aimed to identify compounds that directly inhibit the LEDGF/p75–IN interaction in vitro, only a subset of ALLINIs exhibited this activity. Rather, a shared and defining feature of this class is to act as molecular glues, inducing aberrant hyper‐multimerization of HIV‐1 IN and ultimately impairing viral infectivity.

Reflecting the evolving understanding of their biological behavior, these compounds have been described using various acronyms, including LEDGIN (LEDGF/p75–IN interaction site), NCINI (noncatalytic IN inhibitor), INLAI (IN–LEDGF/p75 allosteric inhibitor), and MINI (multimerization IN inhibitor). Importantly, compounds initially designed as disruptors of the LEDGF/p75–IN interaction were later shown to induce hyper‐multimerization of HIV‐1 IN and to primarily inhibit viral maturation and morphogenesis, rather than the integration step itself. In the following sections, we summarize the key advances in the discovery and development of ALLINIs, highlighting both mechanistic insights and the most promising candidates emerging for the treatment of HIV‐1.

### 2.1 Discovery and Development of Early ALLINIs

Two main strategies were pursued in the search for inhibitors targeting IN through mechanisms different from INSTIs: (i) a structure‐based drug design approach to identify protein–protein interaction inhibitors as disruptors of the LEDGF/p75–IN binding and (ii) high‐throughput screening to identify 3′‐P inhibitors. Following identification, the compounds were characterized to elucidate their multimodal mechanism of action and specific antiviral effects. In this framework, continuous efforts have led to the identification of potent inhibitors that demonstrated efficacy in in vitro models of infection. In addition, investigation of the chemical space through SAR studies allowed for the optimization of specific chemical features of early inhibitors, leading to the identification of additional modulators with improved antiviral potency, PK properties, and resistance profiles, thereby prompting their evaluation as innovative potential drug candidates.

### 2.2 Toward the Search for HIV‐1 IN–LEDGF/p75 Interaction Inhibitors: The Discovery of LEDGINs

Early ALLINI development aimed to block the HIV‐1 IN–LEDGF/p75 interaction using inhibitors that target the IN CCD dimer interface. While peptide derivatives demonstrated that targeting this protein–protein interaction was a feasible endeavor, more prominent efforts have focused on small‐molecule inhibitors owing to their superior potential as therapeutic agents [42, 45, 78, 94, 95, 96].

**FIGURE 6:**
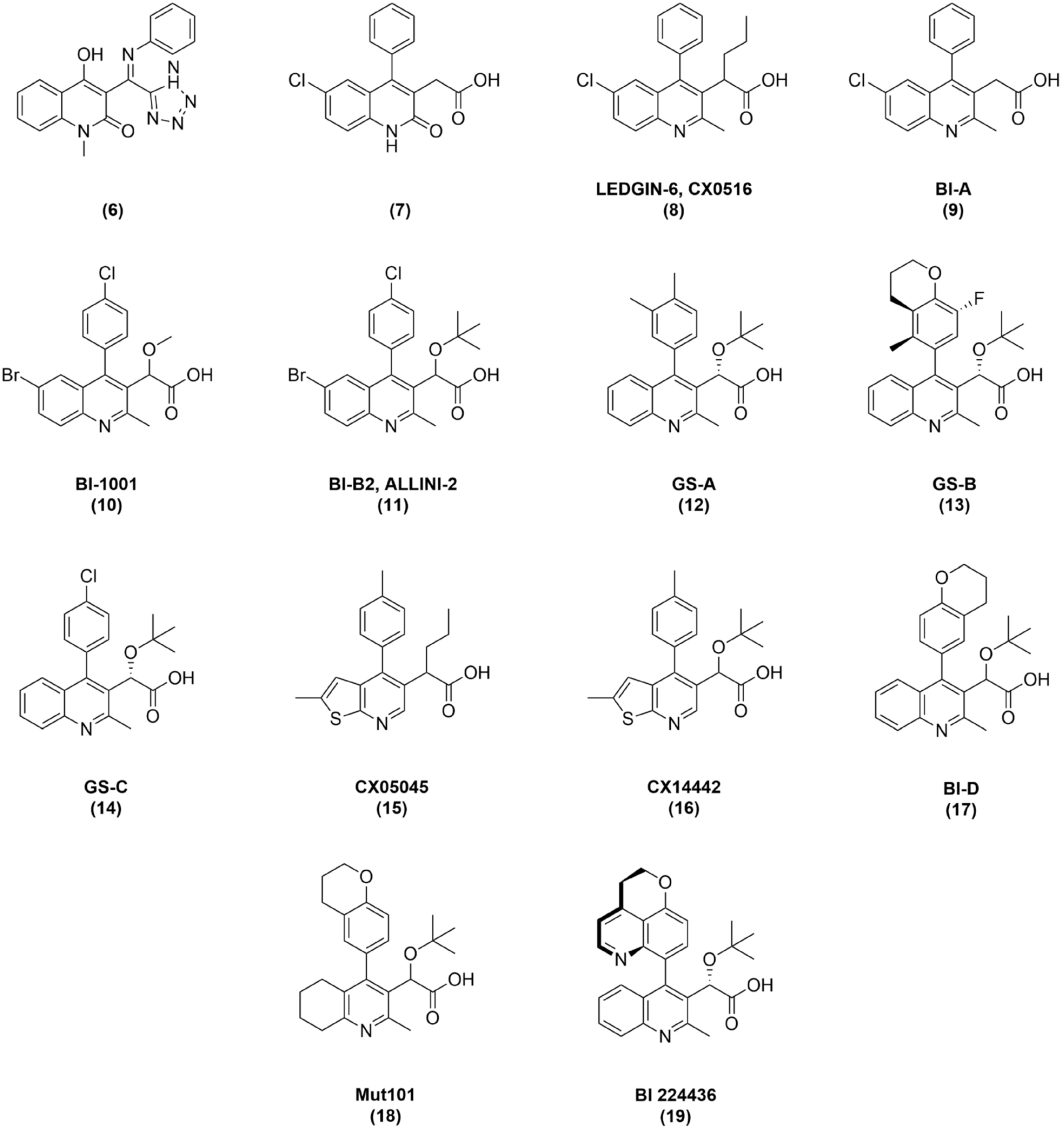
Chemical structures of ALLINIs **6**–**19**.

Building on emerging structural insights into the LEDGF/p75–IN interface [38, 82], structure‐based and virtual screening approaches ultimately led to the identification of the first quinoline‐based ALLINIs, providing early evidence that small molecules could effectively target this interface and exert antiviral effects [79, 97]. In a pioneering study, a large‐scale virtual screening campaign was carried out to derive a consensus pharmacophore model informed by the available crystal structures of the IN CCD [38, 79, 93, 98]. Among the identified hits, the ketimine derivative **6** [79] (Figure 6) was experimentally validated as an in vitro inhibitor of the IN–LEDGF/p75 interaction in the AlphaScreen‐based protein–protein binding assay (Table 1). Beyond eliminating the unstable secondary ketimine at the 3‐position, replacing the tetrazole with a carboxylic acid bioisostere led to the identification of the arylacetic acid **7** [79] (Figure 6) as a more potent inhibitor of LEDGF/p75–IN interaction with moderate antiviral activity and poor cytotoxicity (Table 1).

**TABLE 1:** Biological activity of inhibitors **6**–**19**.

Compounds	**IN–LEDGF binding** **(IC**_**50**_ **, µM)** a	**3′‐P** **(IC**_**50**_ **,** **µM)** b	**ST** **(IC**_**50**_ **, µM)** c	**Antiviral efficacy (EC**_**50**_ **, μM)** d	**CC**_**50**_ **(MT‐4, μM)** e	References
**6**	36%^f^	ND	ND	ND	ND	[79]
**7**	12.2 ± 3.4	ND	ND	41.9 ± 1.1 (MT‐4)	>150	[79]
**8**	10.0 ± 0.4	3.9 ± 0.5	4.2 ± 0.6	12.2 ± 2.9 (SupT1)	59.8 ± 0.5	[79, 99]
**9**	ND	9.0	ND	>40 (C8166)	ND	[100]
**10**	1.0 ± 0.1	2.3 ± 0.1	1.7 ± 0.1	5.8 ± 0.1 (SupT1)	>100	[99, 101]
**11**	0.3 ± 0.02	0.18 ± 0.02	0.17 ± 0.03	0.63 ± 0.30 (HEK293T)	ND	[91]
**12**	0.215	0.106 ± 0.005	0.055 ± 0.003	0.039 ± 0.011 (MT‐4)	76 ± 12	[102]
**13**	0.019	0.151 ± 0.012	0.067 ± 0.004	0.018 ± 0.001 (MT‐4)	42 ± 10	[102]
**14**	0.228	ND	ND	0.055 ± 0.012 (MT‐4)	48 ± 2.5	[102]
**15**	0.58 ± 0.30	ND	ND	0.76 ± 0.08 (MT‐4)	72.2 ± 5.15	[103]
**16**	0.046 ± 0.012	0.739	0.573	0.069 ± 0.003 (MT‐4)	96.0 ± 16.0	[103]
**17**	0.050 ± 0.002	ND	ND	0.19 ± 0.06 (MT‐4)	68 ± 8	[104]
**18**	0.20	ND	0.17	0.54 (MT‐4)	>50	[105]
**19**	0.096 ± 0.003	ND	ND	0.046 ± 0.016 (MT‐4)	>200	[104]

Abbreviation: ND, not determined.

a Concentration required to inhibit the in vitro IN–LEDGF/p75 interaction by 50%.

b Concentration required to inhibit the in vitro 3′‐processing catalytic activity of IN by 50%.

c Concentration required to inhibit the in vitro strand‐transfer catalytic activity of IN by 50%.

d Effective concentration required to reduce HIV‐1‐induced cytopathic effect by 50%; cell lines are indicated in brackets.

e Cytotoxic concentration reducing MT‐4 cell viability by 50%.

f Percent inhibition at 100 μM of the compound.

**FIGURE 7:**
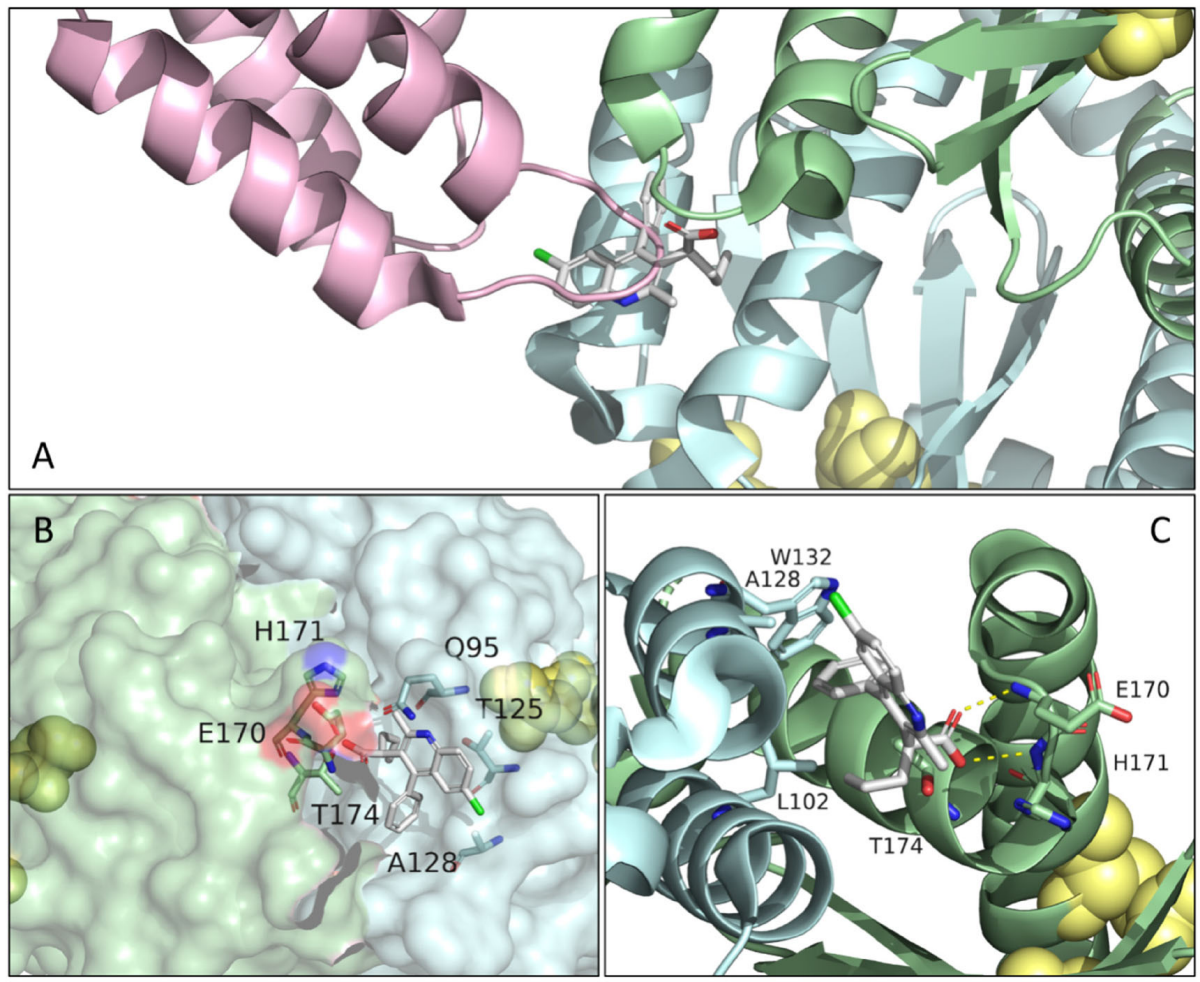
Crystal structure of derivative **8** in complex with LEDGF/p75 binding pocket of the IN CCD dimer (PDB: 3LPU). (A) Cartoon representation of the CCD IN dimer (PDB: 3LPU) in complex with compound **8** superimposed with the IBD‐IN core complex structure (PDB: 2B4J) shown in pink. (B) Co‐crystal structure of compound **8** bound to the IN CCD dimer shown as molecular surface. (C) Focus on compound **8**–IN interactions. Individual IN subunits are depicted in light green and light cyan, compound **8** (white) and the key IN CCD residues (light green and light cyan) are represented as sticks, with nitrogen, oxygen, and chlorine atoms highlighted in blue, red, and green, respectively; the catalytic triad residues are shown as yellow spheres.

Subsequent structure‐based hit‐to‐lead optimization efforts focused on recapitulating the key interactions established by the LEDGF/p75 IBD hotspot residues I365, D366, and L368 at the IN CCD dimer interface (Figure 5), ultimately leading to the identification of the 2‐(quinolin‐3‐yl)acetic acid derivative LEDGIN‐6 [79] (CX0516, **8**, Figure 6). Notably, the compound demonstrated superior potency in inhibiting IN–LEDGF/p75 interaction and enhanced antiviral activity compared to derivative **7** (**8**: EC_50_ = 2.35 µM in MT‐4 cells) [79], while further studies indicated potency against 3′‐P and ST [88, 99] (Table 1). In addition to this, compound **8** exhibited antiviral activity against primary HIV‐1 isolates derived from human donors without cross‐resistance with established antiretroviral drugs, such as RAL, supporting a distinct mechanism of action. In contrast, a viral variant bearing the double mutation A128T/E170G in IN residues forming the LEDGF/p75‐binding pocket, previously selected for resistance to transdominant inhibition by overexpressed LEDGF/p75 IBD [85], was fully resistant to compound **8**, while retaining susceptibility to other antiretroviral agents. Notably, the emergence of the A128T mutation under prolonged selective pressure with **8** strongly supported its function as an allosteric inhibitor that disrupts the LEDGF/p75–IN interaction, as this residue is positioned directly at the protein–protein interaction interface. The co‐crystal structure of derivative **8** in complex with the IN CCD (PDB: 3LPU) showed that the compound occupies the LEDGF/p75‐binding pocket, interacting with the IN residues E170 and H171 via its carboxyl group and reproducing key protein–protein contacts normally mediated by the IBD residue D366 (Figure 7). Furthermore, the phenyl moiety and the chlorine function of **8** nestle in the W132/L102 pocket, clearly mimicking the hydrophobic residues of IBD I365 and L368, respectively (Figure 5). Based on these structural and functional characteristics, compound **8** can be regarded as the prototype of the LEDGIN, representing the first class of ALLINIs specifically optimized to disrupt the LEDGF/p75–IN protein–protein interaction and to produce antiviral effects [79].

### 2.3 Initial Insights Into the Multimodal Mode of Action of ALLINIs

In the search for new antiviral agents, Boehringer Ingelheim reported a series of quinoline‐based inhibitors identified through a high‐throughput screen targeting IN‐catalyzed 3′‐P [106].

The initial hit, referred to as BI‐A [100] (**9**, Figure 6), inhibited IN 3′‐P activity in vitro with micromolar potency, but lacked antiviral activity [100] (Table 1). Subsequent optimization led to more potent analogs, including derivative BI‐1001 [99] (ALLINI‐1, **10,** Figure 6), which exhibited enhanced 3′‐P inhibition and antiviral efficacy [97, 107].

To gain insights into their mechanism of action, derivative **8** was compared with compound **10** [99]. Both compounds inhibited the IN–LEDGF binding and the LEDGF‐independent catalytic activities of IN demonstrating antiviral efficacy in infected cells, with derivative **10** proving more active than compound **8** (Table 1).

X‐ray crystal structures of the compounds bound to the IN CCD dimer revealed nearly overlapping binding modes, indicating that the substituted benzene rings mainly form hydrophobic contacts with one IN monomer, while the carboxyl groups establish hydrogen bonds with residues E170 and H171 of the second monomer (Figure 5). Furthermore, compound **10** was found to establish an extra hydrogen bond between its methoxy group and residue T174, potentially explaining its greater potency compared with compound **8** in both LEDGF‐dependent and LEDGF‐independent assays. The strong similarity in binding modes and biological profiles of the two derivatives supported their classification as ALLINIs. Interestingly, further investigations demonstrated that both compounds promote aberrant IN multimerization by stabilizing IN–IN interactions. The resulting multimers are unable to form the SSC, leading to loss of catalytic activity and impaired downstream LEDGF binding. These findings highlighted a multimodal mechanism for ALLINIs, in which IN multimerization represents the primary event, while disruption of IN–LEDGF interaction and inhibition of catalytic activity are likely secondary consequences, supporting IN multimerization as a promising strategy for the development of ALLINIs [97, 99].

To investigate the structural and mechanistic properties of A128T IN resistance to ALLINIs, derivative **10** was examined alongside its *tert*‐butyl analog BI‐B2 [91] (ALLINI‐2, **11**, Figure 6). Substituting the methoxy group with a *tert*‐butoxy moiety increased thinhibitory potency against 3′‐P and ST, as well as antiviral efficacy, by nearly 10‐fold. Furthermore, this modification tripled the compound's activity in inhibiting the IN–LEDGF/p75 interaction (Table 1). Conversely, the A128T substitution of IN conferred resistance to **11** compared to the wild‐type (WT) virus (**11**: EC_50_ = 12.2 µM); in addition, while the compound exhibited remarkably lower potency in inhibiting the 3′‐P and ST reactions, its impact on the IN–LEDGF/p75 interaction was less pronounced, resulting in only a twofold increase in IC_50_ value. In contrast, a more marked effect was highlighted on IN multimerization, and the A128T substitution was found to produce enhanced resistance to ALLINI‐induced aberrant multimerization of IN compared with WT IN, evidenced by a nearly 10‐fold increase in the IC_50_ values. To elucidate the structural basis of resistance, the crystal structures of **10** bound to either WT or A128T IN CCDs were compared. Interestingly, although the interaction networks between the inhibitor and the protein were largely preserved, the alanine‐to‐threonine substitution was found to affect the positioning of the quinoline core and the 4‐substituted phenyl ring of **10,** likely accounting for the differential multimerization of WT and mutant IN [91].

In this framework, a series of 2‐(*tert*‐butoxy)‐2‐(4‐phenylquinolin‐3‐yl)acetic acids (tBPQAs), originally designed by Boehringer Ingelheim [107, 108] and subsequently licensed to Gilead Sciences, was explored [88, 102]. Compounds GS‐A, GS‐B, and GS‐C [102] (**12**–**14**, Figure 6) displayed remarkable antiviral efficacy in cellular assays, with EC_50_ values ranging from submicromolar to nanomolar concentrations (18 nM < EC_50_ < 55 nM, Table 1) in MT‐4 cells, with compound **13** emerging as the most potent derivative. In resistance selection experiments, compounds **12** and **13** promoted the emergence of mutations clustered at or near the IN dimer interface, such as A128T and T174I. Notably, the resistant viral strains showed cross‐resistance within the tBPQA series but retained susceptibility to RAL, supporting the conclusion that tBPQAs work through a mechanism distinct from that of classical INSTIs. Co‐crystal structures of the IN CCD in complex with compounds **12** and **13** (PDBs: 4E1M and 4E1N) revealed that these molecules bind at the CCD dimer interface, occupying the same pocket targeted by the LEDGF/p75 IBD. Notably, most of the residues identified as mutated in resistance selection studies are located within the inhibitor‐binding site. Specifically, A128 lies close to the quinoline scaffold, while T174 interacts with the carboxylate, *tert*‐butyl, and 4‐phenyl substituents of the ligands.

Investigations into their mode of action indicated that while the derivatives proved to inhibit HIV‐1 integration by elevating the level of 2‐LTR circles and decreasing the number of integration junctions in HIV‐1‐infected cells, the 3′‐P and ST steps were ruled out as targets of inhibition. Instead, the compounds proved to compete with LEDGF for binding to IN dimers, highlighting nanomolar potency consistent with their antiviral EC_50_ values. In addition, similar concentrations were found to promote IN dimer formation, suggesting that these specific interactions underlie the antiviral effects of tBPQAs. In addition to being one of the first reports of ALLINIs achieving antiviral efficacy in the nanomolar range, this study crucially provided innovative insights into the multimodal mode of action of ALLINIs [102].

In the search for new ALLINIs featuring alternative scaffolds, the quinoline moiety was modified into a thienopyridine core, and two analogs of derivative **8** were reported [103]. While derivative CX05045 [103] (**15**, Figure 6) retains the 2‐propyl group of **8**, compound CX14442 [103] (**16**, Figure 6) features a 2‐*tert*‐butoxy moiety, a key hallmark of tBPQAs, to optimize the occupancy of the hydrophobic binding pocket and specifically mimic the IBD residue I365. Interestingly, both thienopyridines proved more active than the quinoline‐based analog, with derivative **16** exhibiting a 10‐fold increase in potency over **15** regarding both LEDGF/p75–IN interaction inhibition and antiviral efficacy [103] (Table 1).

To pinpoint the stage of the virus life cycle affected by derivative **16**, time‐of‐addition (TOA) experiments were performed, obtaining a profile consistent with INSTIs which indicated interference with integration. However, unlike INSTIs, the compound displayed submicromolar activity against both the 3′‐P and ST reactions. Additional studies showed that compound **16** also influences the IN multimerization, by stabilizing the interaction between two IN monomers to likely limit the dynamic rearrangements required for viral DNA binding and thereby impair the intasome assembly. Interestingly, functional studies proved that cells treated with **16** produced noninfectious viral particles, highlighting a long‐term impact of ALLINIs on viral replication. Moreover, the compound displayed potential synergy without cross‐resistance with INSTIs, and no antagonistic effect was observed in combination with RAL [103].

To explore the contribution of LEDGF/p75 to the antiviral activity of ALLINIs, the potency of the 4‐(chroman‐6‐yl)quinoline ALLINI BI‐D [100, 108] (**17**, Figure 6) was explored in WT and *Psip1* (i.e., the gene encoding for LEDGF/p75) KO mouse embryonic fibroblast cells [109]. Interestingly, the depletion of LEDGF/p75 from target cells significantly increased the antiviral activity of the compound, with the IC_50_ decreasing from 2.9 μM in WT cells to 0.16 μM in *Psip1* KO cells. This finding suggested that LEDGF/p75 can compete with the ALLINI for binding to IN during the acute phase of HIV‐1 infection. However, the gain in potency observed upon the removal of LEDGF/p75 importantly raised questions regarding the relevance of the IN–LEDGF/p75 interaction in determining compound potency, indicating that this interaction is not a critical drug target for the compound [109].

Additional key insights emerged by comparing the overall antiviral activity of ALLINIs **10** and **17** with their effects on the early and late phases of the HIV‐1 life cycle [110]. Strikingly, despite its efficacy in a spreading HIV‐1 replication assay (EC_50_ of 5.8 μM), compound **10** did not show an antiviral effect on the early phase of infection (EC_50_ > 50 μM), while demonstrating potency in virus producer cells (EC_50_ of 1.9 μM). Similarly, compound **17** exhibited significantly higher potency in virus producer cells than in target cells, displaying an EC_50_ value that closely matched the value determined in the spreading replication assay (EC_50_ in spreading replication assay = 90 nM; EC_50_ in producer cells = 89 nM), indicating that compound **17** potency is principally accounted for during the late phase of HIV‐1 replication. In addition, analysis of virion morphology by thin‐section electron microscopy revealed that ALLINI treatment significantly enhanced the formation of eccentric HIV‐1 cores, indicating an underlying effect on viral core maturation.

Furthermore, to address if LEDGF/p75 affects **17** potency, the antiviral efficacy of the compound was evaluated in LEDGF/p75‐KO versus control HEK293T target cells. Consistent with previous findings [109], LEDGF/p75‐KO in target cells yielded a significant increase in compound potency. In contrast, LEDGF/p75 depletion did not affect the antiviral potency of the compound in virus producer cells [110].

These findings fundamentally provided key insights into the underlying mechanism of ALLINI action, highlighting that their potency is mainly due to their effects on the late stage of HIV‐1 replication, rather than solely on the inhibition of the IN–LEDGF/p75 interaction during the early stage, culminating in virion morphological defects.

### 2.4 Unraveling the Antiviral Effects of NCINIs and INLAIs

Further insights into the underlying mechanism of action of ALLINIs emerged from investigations on tBPQAs **12** and **13** (Figure 6). Owing to their dual mode of action, comprising the disruption of IN–LEDGF/p75 interaction and the IN dimer promotion, these compounds have been categorized as NCINIs [111]. A two‐part assay system was used to measure the antiviral potency at the late vs. early phase of the virus life cycle by altering the presence of compounds in cultures of virus producer and target cells. Consistent with previous findings [110], a striking functional divergence from RAL emerged during virological profiling: both compounds showed lower potency during the target cell infection phase compared to full‐cycle assay (**12**: EC_50_ vs. full cycle = 75.7 nM, EC_50_ vs. target cells = 6093 nM; **13**: EC_50_ vs. full cycle = 26.4 nM, EC_50_ vs. target cells = 743.5 nM). Conversely, when exposure was restricted to the virus‐production stage, the antiviral activity closely mirrored the full‐cycle results (**12**: EC_50_ vs. producer cells = 84.1 nM; **13**: EC_50_ vs. producer cells = 39.4 nM). This pronounced sensitivity during the late stages of the HIV‐1 replication cycle, coupled with the observation that the IN dimer‐interface mutation T174I confers resistance to these compounds but not to RAL, corroborated that ALLINIs and INSTIs operate through fundamentally distinct inhibitory mechanisms.

Crucially, experimental evidence demonstrated that these compounds act primarily during virus production and/or maturation, while mature virions remained refractory to these inhibitors. Furthermore, consistent with previous findings [109, 110], the variation of LEDGF/p75 expression levels in the virus producer cells had no discernible effect on either viral infectivity or compound potency. This finding indicated not only that LEDGF in producer cells is not essential for HIV‐1 infectivity, but also that the late‐stage inhibitory effect of these compounds is independent of LEDGF. Further investigations clarified that these derivatives do not interfere with Gag/Gag‐Pol processing, RT activity, or viral entry. Instead, WT virions produced in the presence of **13** displayed defective morphological defects in core and reduced infectivity, while the T174I mutation was found to revert such effects. Additionally, **12** was found to affect the oligomeric state of IN, favoring a higher proportion of IN dimers in newly produced viruses. Conversely, no such shifts were observed upon incubating mature, cell‐free virus with the compound, further corroborating the major effect of the compound on viral morphogenesis [111].

In‐depth analyses were performed to dissect particle maturation as the main step underlying the potency of quinoline‐based ALLINIs **11** and **17** [112]. Consistent with previous findings [110, 111], virus produced in the presence of the compounds displayed marked impairment of capsid morphogenesis with a high proportion of virions exhibiting “eccentric” condensates (i.e., electron‐dense aggregates located outside the capsid core). Moreover, the effect of drug treatment on reverse transcription activity closely paralleled the inhibition of mature core formation, and in turn the overall antiviral activity, indicating that their antiviral effect is due to inhibition of particle maturation. Cryoelectron tomography was used to characterize ALLINI‐treated particles, revealing important morphological differences compared to WT. Indeed, “eccentric” condensate‐containing virions from ALLINI treatment displayed higher incidence of nonconical than conical cores, indicating impairment of capsid morphogenesis. Moreover, most of the conical cores appeared relatively empty, revealing deficient incorporation of the viral ribonucleoprotein complex (RNP, assumed to be composed primarily of viral RNA, vRNA, and nucleocapsid protein NC) into the mature core, leaving it to remain outside the capsid shell. Additionally, “tomo‐bubblegram” imaging indicated that “eccentric” condensates have a high NC content, confirming that they represent nonpackaged RNPs [112].

Furthermore, to assess the role of IN in HIV‐1 maturation and the impact of ALLINIs, IN‐deficient particles were transcomplemented with IN fused to the viral accessory protein Vpr. This approach partially rescued the fraction of virions harboring WT cores with internal electron density, while this rescue effect was abolished by ALLINI treatment. Moreover, the genomic vRNA was found to be necessary for ALLINI‐induced eccentric condensate formation, as the fraction of virions exhibiting “eccentric” aggregates did not change upon ALLINI treatment when transfected with RNA‐packaging‐deficient constructs. Overall, these findings demonstrated that IN plays a direct role in initiating core morphogenesis and incorporating the RNP into the mature core during HIV‐1 maturation, in addition to critically suggesting the relevance of the IN–vRNA interaction for core morphogenesis [112].

Additional important findings emerged from investigations on a distinct series of aryl and heteroaryl 2‐(*tert*‐butoxy)acetic acid derivatives referred to as INLAIs [105]. The compounds were found to disrupt the interaction between the IN CCD and the LEDGF IBD as well as the interaction of full‐length LEDGF with IN. Consistently, the derivatives demonstrated significant antiviral activity, which closely correlated with their ability to disrupt IN–LEDGF interactions at submicromolar concentrations (Table 1). Among the series, Mut101 [105] (**18**, Figure 6) emerged as the most effective derivative, acting as the strongest inhibitor of IN–LEDGF binding and displaying the highest antiviral potency. Compound **18** was also able to interfere with LEDGF‐independent IN function, through inhibition of the ST reaction at concentrations comparable to those disrupting the IN–LEDGF interaction, despite resulting less potent than RAL. Co‐crystal structure of compound **18** bound to the IN CCD dimer (PDB: 4LH5) showed that it engages the LEDGF‐binding pocket, establishing interactions consistent with those reported for previous inhibitors.

However, unlike previously reported ALLINIs, compound **18** and the related quinoline derivative **17** (Figure 6) retained full antiretroviral activity against the HIV‐1 mutant bearing the IN A128T mutation, and the compounds proved to stabilize both the WT and mutant enzyme in a tetrameric form. In addition, compound **18** showed efficacy against a broad panel of drug‐resistant viral strains, including INSTI‐resistant variants. Interestingly, while TOA experiments indicated interference with integration, the compound demonstrated a more potent antiviral effect during the late, postintegration phase of the replication cycle. Differently, the HIV‐1 T174I mutant (i.e., mutated in the LEDGF‐binding pocket) exhibited reduced sensitivity to compound **18**, resulting in the production of infectious virions [105].

Although a postintegration block in virus‐producer cells was found to be the primary mechanism of its antiretroviral activity, the compound's dual effect on both integration and postintegration was attributed to its binding at the LEDGF‐binding pocket of IN. This discrepancy in activity was explained based on the cellular localization of the cofactor: in the nucleus of target cells, LEDGF outcompetes the compound for the IN binding site [109], reducing its efficacy during integration. Conversely, in the cytoplasm (where postintegration assembly of infectious particles occurs) LEDGF is absent, allowing the compound to exert its full effect [105].

### 2.5 Toward the Search of Effective Quinoline‐Based ALLINIs: The Discovery of BI 224436

In the search for effective ALLINIs, extensive hit‐to‐lead optimization efforts were conducted by Boehringer Ingelheim to refine the structural determinants governing activity in quinoline‐based inhibitors. These studies established meaningful SARs within this class of compounds, culminating in the discovery of BI 224436 [96] (**19**, Figure 6), the first ALLINI to advance into Phase IA clinical trials [106, 113].

**FIGURE 8:**
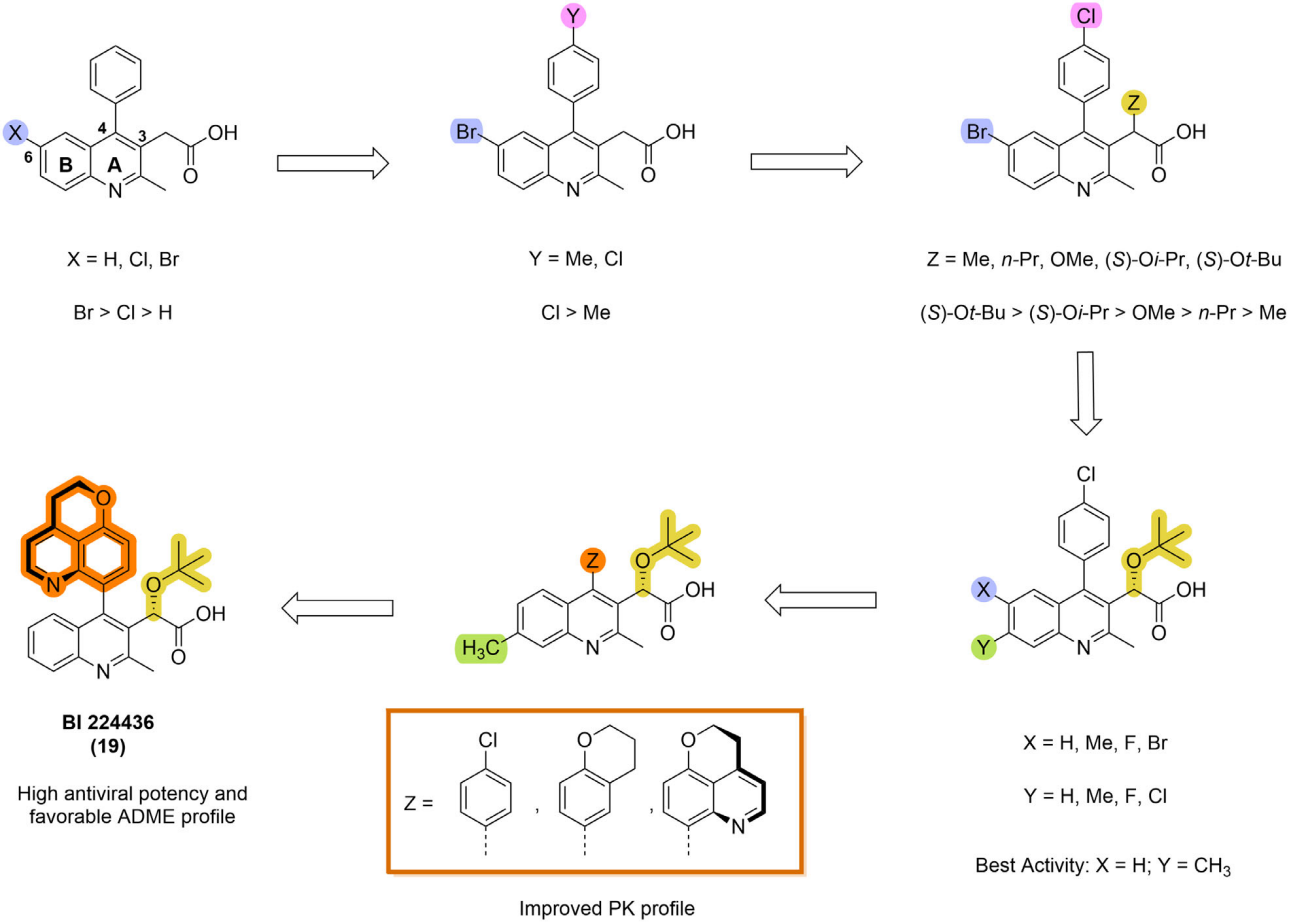
Structural optimization to yield compound **19**.

**FIGURE 9:**
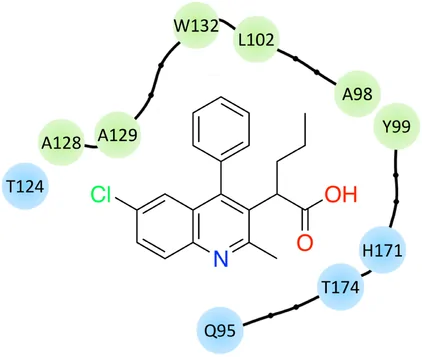
Structural features of ALLINI binding. View of the ALLINI binding pocket and crystallographic organization of IN subunits.

Starting from compound **9** (Figure 6), which was identified as 3′‐P inhibitor through high‐throughput screening, SAR studies were carried out by introducing modifications at three main regions of the molecule: the α‐position of the C3 acetic acid moiety, the arene at C4, and the B‐ring (i.e., the phenyl ring fused to the pyridine moiety of the quinoline scaffold) (Figures 8 and 9).

In addition to exploring 3′‐P inhibition, structural insights from X‐ray crystallography studies were used to optimize interaction networks of the quinoline‐based inhibitor and the IN CCD dimer [79, 91, 99] (Figure 9).

In this framework, early optimization efforts focused on modifications at the 6‐position. While replacing the 6‐chlorine of **9** with a bromine atom improved the potency against 3′‐P, the deschloro analog exhibited lower activity. Additionally, the antiviral efficacy of 6‐bromine‐substituted derivatives was further enhanced by introducing a methyl or a chlorine group at *para*‐position of the 4‐phenyl group. By keeping untouched the 6‐Br and 4‐(*p*‐chlorophenyl) substituents, functionalization at the α‐position of the C3 acetic acid moiety was found to be generally beneficial for potency. While the methyl‐substituted derivative retained comparable activity to the acetic analog, further enlargement of the alkyl substituent led to marked improvements in antiviral activity. In particular, the insertion of a *n*‐propyl group yielded a derivative (i.e., the α‐propyl analog of **10**) displaying potency against 3′‐P (IC_50_ = 0.077 µM in the LTR‐cleavage assay) and antiviral efficacy (EC_50_ = 0.92 µM) at submicromolar concentrations, together with negligible cytotoxicity. Based on these encouraging results, a broader range of α‐substituents was examined, revealing that alkoxy groups could confer additional gains in potency (Figure 8). Given the robust activity highlighted by the methoxy derivative (**10**), the racemic mixture was subsequently resolved into its individual enantiomers, with the *S*‐enantiomer possessing superior intrinsic and antiviral activity than the racemate.

Progressive enlargement of the alkoxy group at the C3 position to establish additional interactions within the target pocket led to the identification of the *tert*‐butoxy group as the optimal substituent. In particular, the *S*‐enantiomer of **11** proved antiviral efficacy in the nanomolar range (EC_50_ = 78 nM) and was used as a starting point for further optimizations [106].

To enhance the antiviral properties and explore additional binding pocket interactions, further derivatives were obtained by preserving the α‐ *tert*‐butoxy and the 4‐(*p*‐chlorophenyl) groups while replacing the 6‐Br with alternative substituents. SAR analysis revealed that the introduction of bulky aryl substituents at this position negatively affected the scaffold's multimerization‐inducing properties, leading to a progressive loss of activity. Similar detrimental effects were observed by inserting substituted phenyl rings and other bulky groups, including pyridinyl, six‐membered nonaromatic heterocycles, and five‐membered aromatic heterocycles [114]. At the same time, X‐ray analysis revealed that removing the 6‐bromine substituent caused a shift in the quinoline scaffold. This repositioned the C5 atom closer to A128 and drove the C4 substituent deeper into the highly conserved region of the binding pocket. Furthermore, derivatives lacking the 6‐bromine atom and carrying a 7‐methyl group showed antiviral efficacy against strains with 124 and 125 polymorphisms, leading to the adoption of these modifications for lead optimization [106].

Modifications to the 4‐position of the quinoline scaffold were also explored to enhance interactions with the hydrophobic pocket formed by residues W132 and L102 of subunit 2 of IN, with the aim of improving the compounds’ multimerization efficacy (Figure 9) [97]. Aromatic substituents at this position were generally favored, with substituted phenyl groups emerging as optimal. A broad exploration of *para*‐substituted phenyl derivatives revealed that incorporation of fluorine or fluorinated groups, such as trifluoromethyl and trifluoromethoxy, led to modest improvements in compound multimerization efficacy, whereas *para*‐methyl or *para*‐methoxy substitutions produced approximately sixfold gains in multimerization activity. The *para*‐chloro analog achieved the most potent inhibition within this series (EC_50_ ~ 100 nM in the multimerization assay), attributed to a stabilizing chlorine–π interaction with W132 (Figure 9). In contrast, alternative *para*‐substituents such as cyano, acetyl, acetamido, or phenyl groups reduced potency, highlighting the steric constraints of the hydrophobic pocket. *Meta*‐ or *ortho*‐substituted phenyl derivatives were less effective than their *para* counterparts because these positions orient the substituents away from the W132/L102 pair, attenuating hydrophobic interactions. For most substituents, the observed potency trend was *para *> *ortho *> *meta*. Attempts to combine *para* and *meta* substituents into disubstituted phenyl groups did not significantly improve compound multimerization efficacy, with activity either similar to that of the *para* isomer or even worse [97].

A more in‐depth analysis of the C4 substituent proved to be the most productive strategy for enhancing antiviral potency, as larger ring systems were generally more effective at occupying the binding pocket than smaller pentadiene‐like heterocycles such as furan or thiophene, thereby leading to increased multimerization potential. In particular, the introduction of a chromane moiety at C4 markedly improved both in vitro IN multimerization and antiviral efficacy in the nanomolar range [97, 106]. Further analogs incorporating related bulky ring systems, such as benzodioxane and substituted chromane motifs, confirmed that larger C4 arenes were particularly effective at filling the conserved binding pocket. Progressive occupation of this region also reduced the fold shift between EC_50_ values measured across IN variants carrying different aa124/aa125 residues, indicating a more robust interaction profile. Mechanistic investigations of the chromane‐substituted derivative confirmed its categorization as NCINI, in line with its ability to interfere with the IN–LEDGF interaction and modulate IN multimerization.

Additionally, introducing substituents that restricted rotation around the C4—C(arene) bond was found to further improve the antiviral potency, as highlighted by stable atropisomers. A key advancement was achieved through the hybridization of chromane and quinoline features, leading to tricyclic C4 arenes that possessed potent antiviral efficacy across IN variants while maintaining favorable metabolic stability (Figure 8). Finally, the removal of the 7‐methyl group of the quinoline scaffold resulted in improved serum shift, excellent metabolic stability, and favorable in vitro ADME profile, giving rise to compound **19** [106] (Figure 8). Interestingly, the derivative exhibited nanomolar antiviral activity against a broad range of mutant viruses, including variants carrying substitutions at the highly polymorphic IN residues 124 and 125 (11 nM < EC_50_ < 27 nM). Notably, given its excellent PK profile in animal models, such as high oral bioavailability and low clearance, compound **19** advanced into Phase I clinical trials, marking a major milestone in the field as the first ALLINI to undergo clinical evaluation [115]. Although its clinical development was later halted for undisclosed reasons, compound **19** has remained an important research tool, contributing significantly to the understanding of the mechanistic basis of ALLINI‐mediated inhibition [116, 117].

**FIGURE 10:**
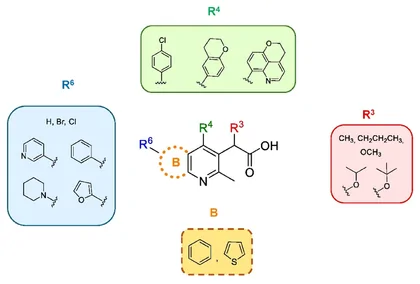
Overview of the main structural modifications introduced on the quinoline scaffold to investigate SARs. Substituents at different positions of the core were systematically varied to assess their impact on IN function, LEDGF–IN interaction, and antiviral activity.

Overall, modifications of the quinoline scaffold, summarized in Figure 10, yielded important insights into the therapeutic relevance of ALLINIs, in addition to serving as key investigational tools to elucidate key mechanistic evidence, as discussed in the following paragraphs.

Nevertheless, SAR studies demonstrate how specific structural features and the fine‐tuning of key substituents have enabled effective interference with the IN–LEDGF interaction, modulation of IN multimerization, and potent antiviral efficacy. The balance between these effects appears to be governed by the nature and steric properties of the substituents, which dictate pocket occupancy and the stabilization of specific IN oligomeric states.

### 2.6 Optimizing ALLINIs for Targeting IN Multimerization

The studies described in the previous sections provided important insights into the mechanism of action of ALLINIs and simultaneously delineated relevant SARs for quinoline‐based inhibitors. Strikingly, although most of these compounds were originally designed to inhibit the LEDGF/p75–IN interaction, the observation that they can induce aberrant IN multimerization, likely the key determinant of their antiviral activity, prompted a more focused investigation of this alternative mechanism in the development of new antiretroviral agents. In this context, derivatives originally designed as putative disruptors of the LEDGF/p75–IN interaction were more extensively evaluated for their potential to induce multimerization of IN. In addition, efforts were devoted to elucidating SARs that relate structural determinants with their inhibitory potency toward LEDGF/p75–IN binding and their efficacy in promoting IN multimerization, while prominent research focused on optimizing IN multimerization as a key determinant of ALLINI antiviral efficacy. Remarkably, these findings were greatly enhanced by structural insights, significantly supporting the understanding of the molecular basis for ALLINI efficacy, as discussed in the following paragraphs.

### 2.7 Pyridine‐Based Inhibitors

**FIGURE 11:**
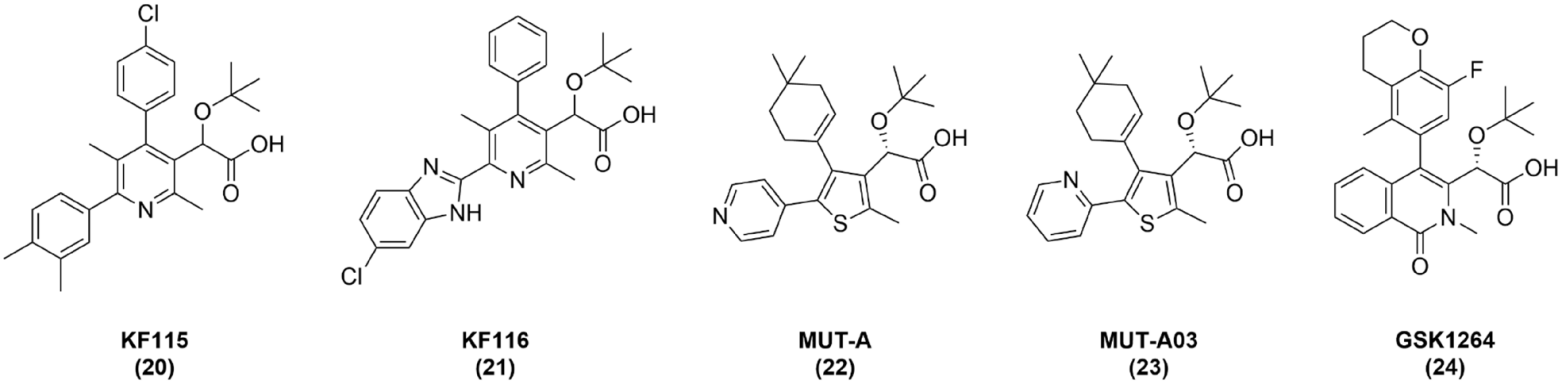
Chemical structure of ALLINIs **20**–**24**.

**FIGURE 12:**
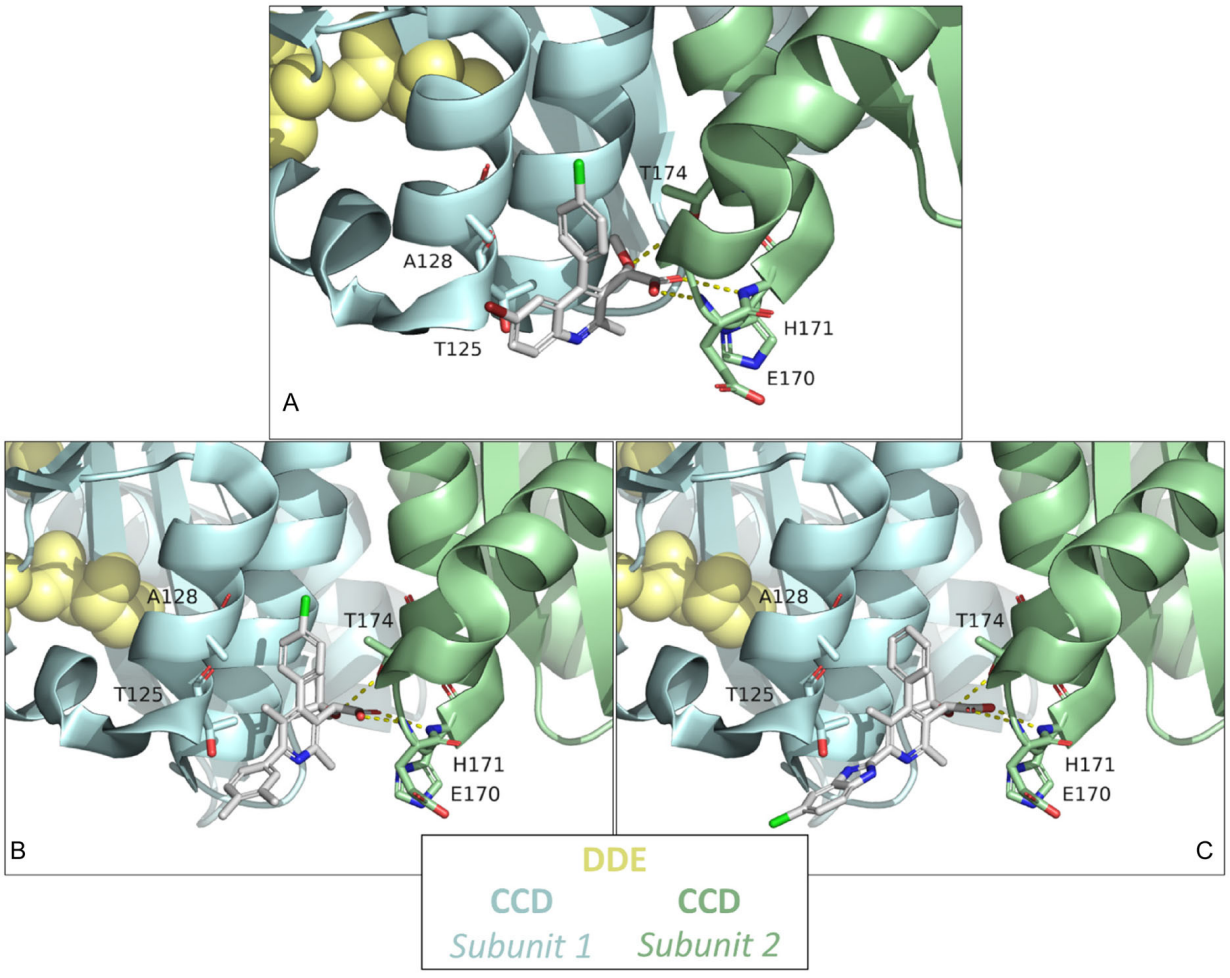
Crystal structure of compounds **10**, **20**, and **21** bound to HIV‐1 IN CCD dimer interface (PDBs: 4DMN, 4O0J, 4O55). Cartoon representation of **10** (A), **20** (B), and **21** (C) bound to the IN CCD dimer. The IN subunit 1 and 2 are colored in light cyan and light green, respectively, compounds are represented as white sticks, with nitrogen, oxygen, bromine, and chlorine atoms highlighted in blue, red, brown, and green, respectively.

To specifically examine the role of HIV‐IN multimerization independently of the IN–LEDGF/p75 interaction, a series of selective multimerization inhibitors, referred to as MINIs, were designed and synthesized (Figure 11) [101]. Structural comparison of the crystal structures of compound **10** (Figure 6) (PDB: 4DMN) and the LEDGF/p75 IBD (PDB: 2B4J) bound to HIV‐1 IN CCD dimers revealed that both ligands occupy the LEDGF/p75 binding pocket and act as molecular bridges between the two IN subunits (Figures 7 and 12A). Specifically, each molecule establishes hydrogen bond interactions with one subunit (subunit 2) through contacts with residues E170 and H171, while the methoxy group of compound **10** further interacts with residue T174. In contrast, interactions with the other subunit (subunit 1) are mainly hydrophobic and appear constrained by the rigid, planar nature of the quinoline core (Figures 7 and 12A). To strengthen contacts with this subunit, the quinoline scaffold was subsequently modified into a pyridine‐based core, leading to the development of derivatives KF115 [101] (**20**, Figure 11) and KF116 [101] (**21**, Figure 11). X‐ray crystallographic analyses of compounds **20** and **21** in complex with the HIV‐1 IN CCD dimer (PDBs: 4O0J, 4O55) showed that, although interactions with subunit 2 are comparable to those observed for compound **10**, hydrophobic contacts with subunit 1 are strengthened, largely due to the increased molecular flexibility of these derivatives. Moreover, the benzimidazole moiety of compound **21** establishes an additional interaction with subunit 1 through a hydrogen bond with residue T125 (Figure 12).

Notably, structural modifications of quinoline **10** significantly altered the biological profiles of the pyridine‐based compounds. In particular, the new derivatives exhibited higher potency and selectivity (up to 60‐fold) toward inducing IN multimerization over disrupting the LEDGF/p75–IN interaction (Table 2), in contrast with earlier ALLINIs like **10**, which displayed comparable activity toward both processes (**10**: EC_50_ for aberrant IN multimerization = 4.9 µM; IC_50_ for IN–LEDGF/p75 binding = 1.0 µM) [101]. Interestingly, the compounds showed antiviral effects at concentrations similar to those inducing IN multimerization, suggesting that such event, rather than the inhibition of the LEDGF/p75–IN interaction, drives their efficacy (Table 2).

**TABLE 2:** Biological activity of compounds **20**–**24**.

Compounds	**IC**_**50**_ **IN–LEDGF binding,** **μM** a	**EC**_**50**_ **for aberrant IN multimerization,** **μM** b	MT‐4		References
			**Antiviral efficacy (EC**_**50**_) **μM** c	**CC**_**50**_ **,** **μM** d	
**20**	13.0 ± 1.5	0.274 ± 0.025	0.121 ± 0.004	>100	[101]
**21**	5.03 ± 0.36	0.086 ± 0.006	0.024 ± 0.003	>100	[101]
**22**	0.095 ± 0.005	0.052 ± 0.003^e^	0.031 ± 0.009	42 ± 9	[104]
**23**	0.19	0.37^e^	0.45	ND	[104]
**24**	0.0082 ± 0.00014^f^	ND	0.038	ND	[118]

Abbreviation: ND, not determined.

a Concentration required to inhibit the in vitro IN‐LEDGF/p75 interaction by 50%.

b Concentration required to induce IN multimerization of IN by 50%.

c Effective concentration required to reduce HIV‐1‐induced cytopathic effect by 50% in MT‐4 cells.

d Cytotoxic concentration reducing cell viability by 50% in MT‐4 cells.

e Expressed as activation concentration (AC_50_).

f Inhibition of LEDGF/p75 IBD–IN^F185K^ CCD binding expressed as pIC_50_.

While compound **21** proved more potent than **20** (Table 2), its antiviral efficacy was attenuated against the HIV‐1 IN T125A mutant, validating the functional relevance of the hydrogen bond established with the benzimidazole moiety (Figure 12). In contrast, the compound proved to maintain efficacy against the challenging HIV‐1 IN A128T mutation, effectively promoting IN multimerization and impairing HIV‐1 replication despite this substitution. While such mutation typically introduces steric and electronic constraints that hinder the binding of quinoline‐based ALLINIs (e.g., derivative **10**) [91], the alternative orientation of the benzimidazole moiety of compound **21** allows it to successfully circumvent these steric effects. Consistent with previously reported ALLINIs [105, 110, 111, 112], compound **21** was more effective in producer than target cells (EC_50_ vs. target cells = 50.9 µM; EC_50_ vs. producer cells = 30 nM) at concentrations similar to those seen in the full replication cycle (EC_50_ = 24 nM). In addition, morphological analysis of producer cells treated with **21** revealed an impaired formation of electron‐dense cores, resulting in the production of virions characterized by “eccentric” cores lacking RNPs [101, 112]. Further experiments indicated that the binding of **21** to the IN CCD dimer interface stabilizes the IN subunits, effectively shifting the equilibrium toward aberrant, higher‐order oligomerization.

Interestingly, comparing the antiviral profiles of **21** and the quinoline‐based ALLINI **13** [102] revealed that while both compounds primarily target the late stage of viral replication, **21** is a more potent postintegration inhibitor. In contrast, **13** displayed a more pronounced effect on target cells versus producer cells compared to **21** (**13**: IC_50_ vs. target cells ~0.7 µM; **21**: IC_50_ vs. target cells > 50 µM), which notably aligns with its higher potency in disrupting the IN–LEDGF/p75 binding relative to the pyridine‐based inhibitor (Tables 1 and 2).

Overall, these findings highlighted important divergent functional features for the two classes of compounds, indicating that the superior antiviral efficacy of the pyridine derivatives stems from their capacity to induce IN multimerization rather than interfering with LEDGF/p75 binding, consistent with their higher potency in producer cells.

In addition to compound **21**, other pyridine‐based analogs have been reported as potential ALLINIs, showing improved drug‐like characteristics compared with quinoline derivatives in ADME and in vivo PK studies [119, 120]. Nevertheless, despite these encouraging properties, none of these compounds, including derivative **21**, has advanced to clinical evaluation.

### 2.8 Thiophene‐ and Isoquinolone‐Based Inhibitors

In the pursuit of diversifying the chemical space of ALLINIs, a novel set of thiophene derivatives was identified as structural alternative to established quinoline classes [121]. Optimization of this series, driven by the necessity to maintain the pharmacophoric *tert*‐butoxy acetic acid moiety common to potent ALLINIs, led to the discovery of the lead compound MUT‐A [121] (**22,** Figure 11). This inhibitor, characterized by a five‐membered thiophene ring substituted with methyl, a *gem*‐dimethylcyclohexenyl, and 4‐pyridinyl moieties at positions 2, 4, and 5, respectively, of the central core, demonstrated a potent dual mechanism of action. Biochemically, nanomolar concentrations of **22** effectively disrupted the interaction between IN and LEDGF/p75, while concomitantly inducing the aberrant multimerization of the viral protein. These biochemical properties translated into robust antiretroviral activity, with the compound exhibiting antiviral efficacy within the nanomolar range with negligible cytotoxicity (Table 2). Consistent with previous findings, virions produced in the presence of **22** displayed severe infectivity defects and were noninfectious [112, 121].

**FIGURE 13:**
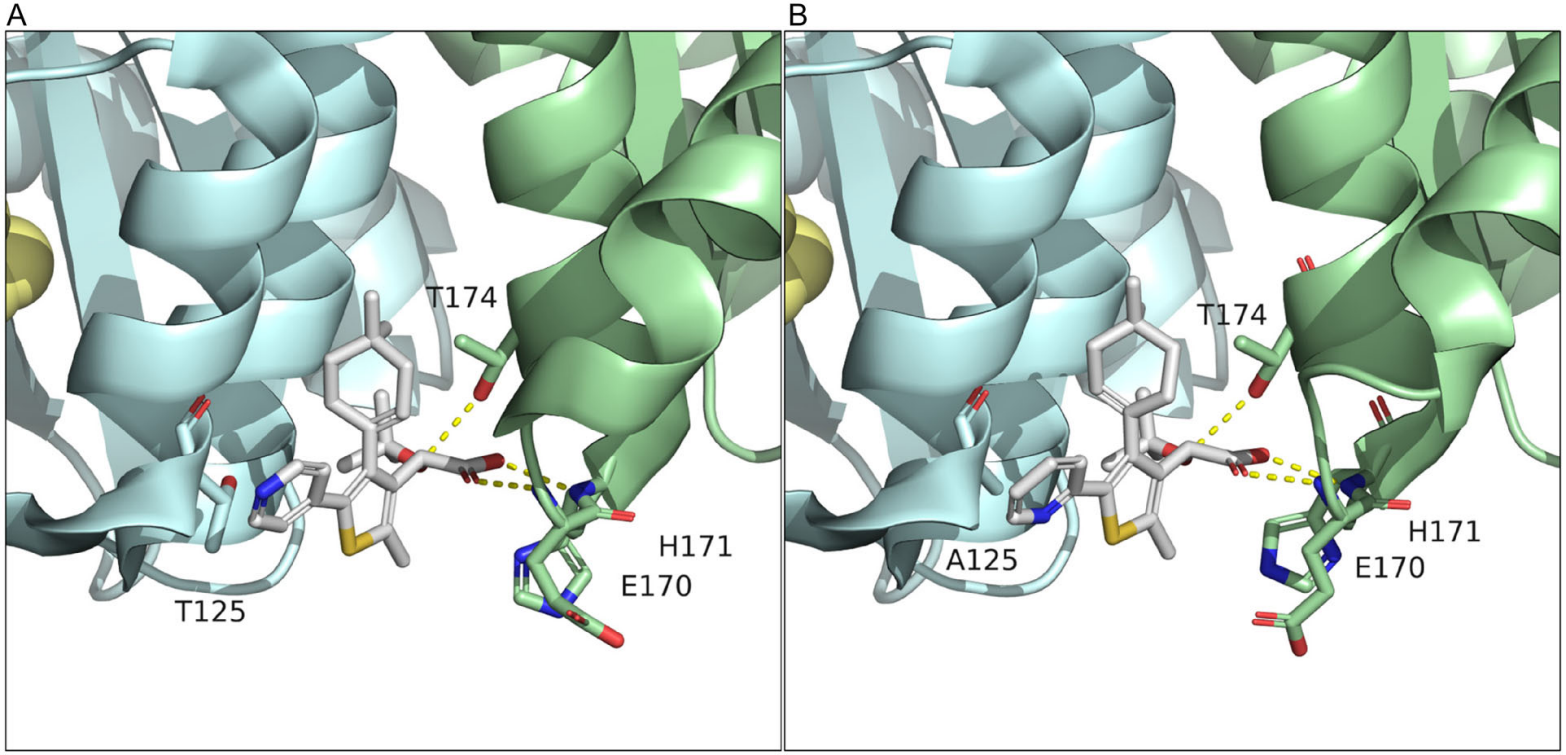
Crystal structures of compounds **22** (A) and **23** (B), bound to HIV‐1 IN CCD dimer interface (PDBs: 5OI2 and 5OI8). CCD dimer chains are represented with cartoons and colored in light cyan and light green, respectively. The catalytic triad residues are shown as yellow spheres. Compounds **22** and **23** are represented as white sticks, with nitrogen, oxygen, and fluorine atoms highlighted in red, blue, and pale cyan, respectively.

However, the therapeutic potential of **22** was challenged by its sensitivity to natural polymorphisms within the IN CCD, especially at the highly variable residues 124 and 125 [104]. Interestingly, detailed virological profiling revealed that while **22** retained potency against variants bearing T125, its antiviral efficacy was drastically reduced, by approximately 50‐fold, against viruses harboring an alanine at this position. Crucially, the latter represents a highly prevalent polymorphic form, particularly within specific viral clades, which may inherently compromise the clinical utility of inhibitors sensitive to this substitution. Indeed, sequence analysis indicates that these A125‐harboring strains are dominant [122, 123]. Mechanistic decoupling studies using the A125 variants revealed a critical insight into ALLINI pharmacology: while **22** retained its ability to bind the allosteric pocket and inhibit the IN–LEDGF/p75 interaction, it completely failed to induce the requisite aberrant multimerization of the A125 IN variant. This observation underscored that affinity for the LEDGF‐binding pocket is necessary but insufficient for the late‐stage antiviral effect, which strictly depends on the induction of specific conformational changes leading to oligomerization. Crystallographic studies of the IN CCD complexed with **22** elucidated the structural basis of these findings. The X‐ray structures (PDBs: 5OI2 and 5OI8) indicated that in the context of the A125 variant, **22** binding stabilized the CCD dimer interface, preventing the formation of the aberrant higher‐order oligomers required for the inhibition of viral maturation. Conversely, in the WT T125, ligand binding destabilized the dimer, facilitating multimerization (Figure 13). Guided by these structural insights, a SAR campaign focused on the substituent at position 5 of the thiophene core was undertaken to restore activity against the A125 variant [104]. This effort led to the identification of MUT‐A03 [104] (**23** Figure 11), an analog where the 4‐pyridyl moiety of **22** was replaced by a 2‐pyridyl group. Structural comparison (PDBs: 5OI2 and 5OI5) showed that unlike **22**, where the pyridine nitrogen is solvent‐exposed, the nitrogen in **23** is buried within the binding site (Figure 13B). Crucially, protein interfaces, surfaces, and assemblies analysis predicted that **23** maintains the ability to destabilize the CCD dimer even in the A125 variant, correlating with its recovered ability to promote IN multimerization and its improved antiviral efficacy against this polymorphic variant compared to the parent compound. Although **23** successfully overcame the specific resistance conferred by the A125 polymorphism, it exhibited a trade‐off in potency against the WT T125 virus, highlighting the delicate structural balance required to engage the allosteric pocket effectively across diverse viral genetic backgrounds. Consequently, although these compounds provided a fundamental proof‐of‐concept (POC) for dissociating the biochemical activities of ALLINIs, their development was ultimately halted due to this inconsistent potency profile across different viral genotypes.

**FIGURE 14:**
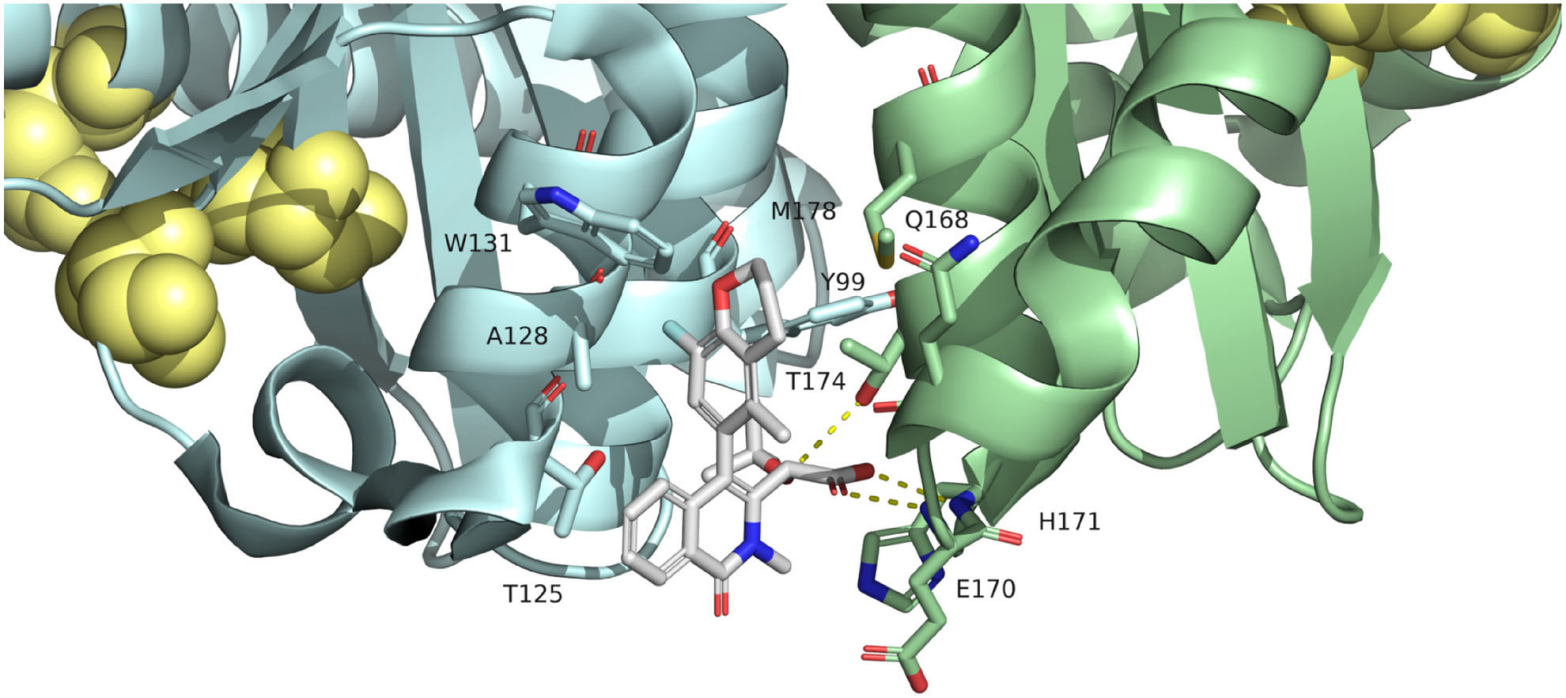
Crystal structure of compound **24** bound to HIV‐1 IN CCD dimer interface (PDB: 4OJR). CCD dimer chains are represented with cartoons and colored in light cyan and light green, respectively. The catalytic triad residues are shown as yellow spheres. Compound **24** is represented as white sticks, with nitrogen, oxygen, and fluorine atoms highlighted in red, blue, and pale cyan, respectively.

While the thiophene‐based series explored the impact of core diversification on polymorphic variants, parallel drug discovery efforts continued to refine the more traditional bicyclic architectures. Indeed, significant advancements were also achieved by modifying the quinoline core into more complex fused systems. A prominent example is the isoquinolinone derivative GSK1264 [118, 124] (**24**, Figure 11). The compound potently disrupted the LEDGF/p75 IBD–IN^F185K^ CCD interaction (IC_50_ ≈ 6.3 nM) and inhibited HIV‐1 replication in MT‐4 cells with an EC_50_ of ~38 nM in a multicycle assay. X‐ray crystallography of **24** bound to the IN CCD dimer interface (PDB: 4OJR) showed occupancy of the LEDGF pocket and engagement of both IN monomers through hydrophobic and polar interactions consistent with other ALLINIs (Figure 14). In these structural studies, the IN^F185K^ variant was employed to improve protein solubility and reduce nonspecific aggregation while preserving functional and binding properties, thereby enabling crystallographic characterization of the inhibitor‐bound complex [125, 126, 127].

As observed for related derivatives, **24** displayed lower antiviral activity during early infection compared with its potency in multicycle and late‐stage assays. Evaluation of LEDGF dependence revealed only a modest increase in activity in LEDGF KO versus WT cells (≈5‐fold), smaller than that reported for quinoline‐based ALLINIs such as **17** (≈30‐fold). Overall, these results suggest that compound **24** is less effective at displacing LEDGF from IN and primarily exerts its antiviral activity by promoting aberrant IN multimerization. In line with this mechanism, the compound induced concentration‐ and time‐dependent IN polymerization, leading to the formation of insoluble aggregates and ultimately impairing the production of mature virions [125].

### 2.9 Key Insights Into the Structural Basis for ALLINI Function

Discovering that not only the CCD, but also the CTD plays a pivotal role in driving IN hyper‐multimerization represented a key step forward in elucidating the underlying mode of action of ALLINIs [128, 129]. Initial molecular modeling studies suggested that these inhibitors directly bridge the interface between a CCD dimer and the CTD of an adjacent dimer. In the absence of an inhibitor, this interface is typically occupied by water molecules, which minimize direct CCD–CTD interactions. However, the binding of an ALLINI effectively fills the V‐shaped pocket, displacing water molecules and enhancing the binding interface for the incoming CTD [130].

**FIGURE 15:**
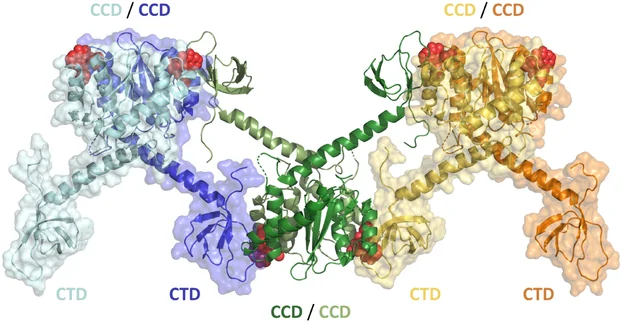
Crystal structure of HIV‐1 IN^Y15A/F185H^ bound to compound **24** (PDB: 5HOT). Cartoon representation of the open polymer formed upon binding of **24**. Each monomer is presented in different colors and compound **24** located at CTD–CCD interfaces is shown as red spheres.

This model was structurally validated using a full‐length HIV‐1 IN^Y15A/F185H^ mutant, specifically engineered to improve solubility while retaining enough drug‐induced aggregation to allow for crystallization [131, 132] and bound to compound **24**. The resulting structure (PDB: 5HOT, recently superseded by PDB: 8V9C [133]) provided a clear visualization of the open polymer architecture, where the inhibitor mediates head (CCD–CCD dimer) to tail (CTD of another dimer) interactions (Figure 15) [125]. This structure indicated that the bound compound **24** enhances and strengthens CCD–CTD interactions, promoting the formation of extended IN polymers. This event leads to aberrant multimerization, resulting in IN aggregation and subsequent enzyme inactivation, thus providing a rational explanation for the underlying mode of action of the compound [13].

Expanding on this concept, further studies [134] demonstrated that these aggregates are not merely linear chains but complex branched polymers with fractal‐like properties. These insights revealed that while the inhibitors stabilize the CCD–CTD junction, the 3D network is further driven by homomeric CTD–CTD interactions, which act as branching points that transform the polymer into an inactive, gel‐like state.

**FIGURE 16:**
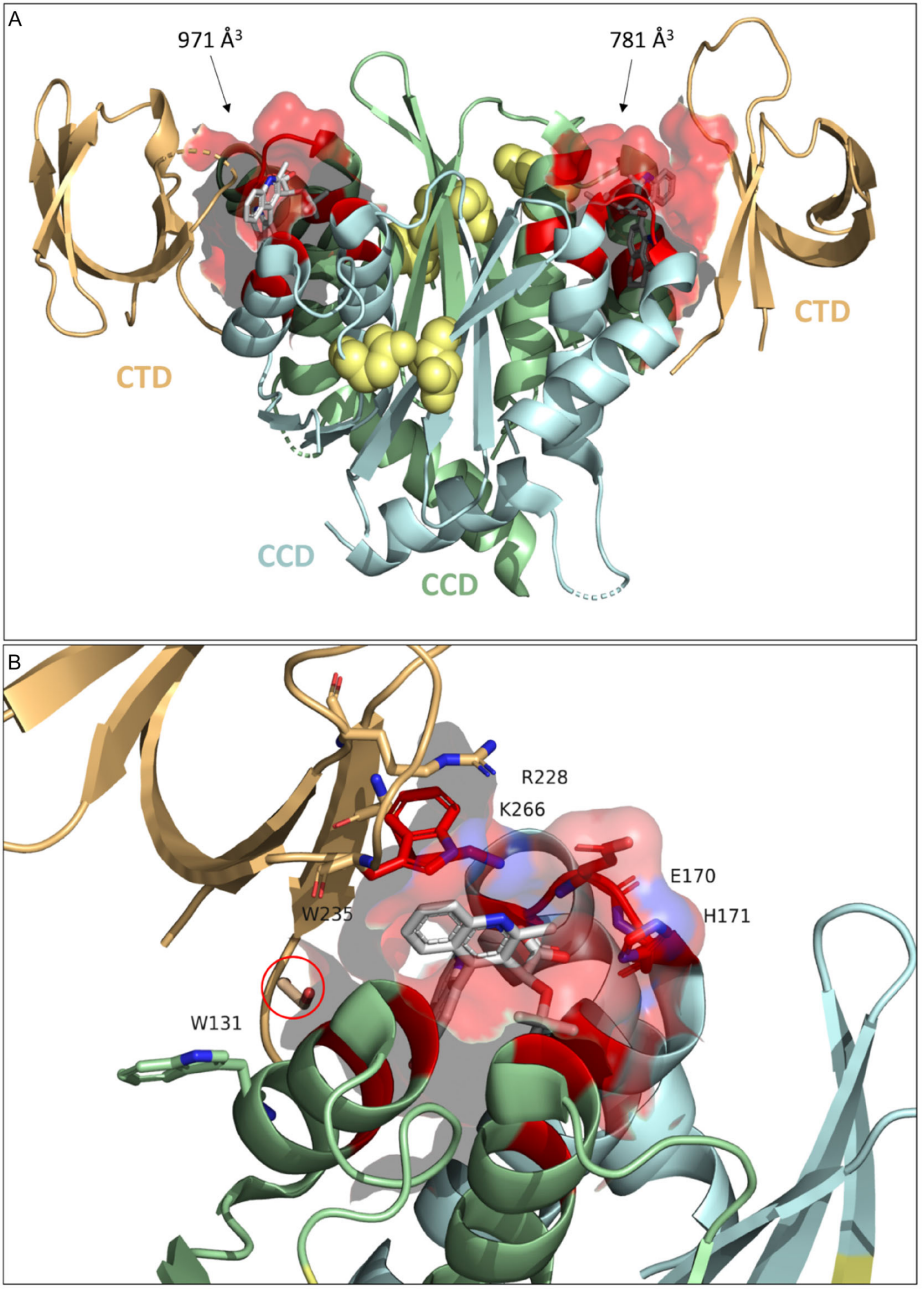
Crystal structure of HIV‐1 IN bound to compound **19** (PDB: 8CTA). (A) Cartoon representation of two molecules of **19** located at CTD–CCD interfaces. (B) Focus on the novel binding pocket identified at the CCD–CTD interface. CCD dimer chains are colored in light cyan and light green, respectively, while CTD is represented in gold. The catalytic triad residues are shown as yellow spheres. Compound **19** is represented as white sticks. The two asymmetric sites are shown in red.

These insightful findings paved the way for further structural investigations, many of which have focused on the quinoline‐based derivative **19** (Figure 6). Indeed, recent high‐resolution crystallographic data (PDB: 8CTA) [116] have refined the understanding of this compound's binding mode, revealing a surprising asymmetry in the ternary complex (Figure 16). The study identified two nonidentical binding sites: a well‐ordered Site 1 (volume ~781 Å^3^) and a more flexible, less compact Site 2 (volume ~971 Å^3^), which exhibits weaker electron density for key residues. Their structure highlighted a dense network of interactions. Specifically, W235 of the CTD provides a favorable π–π interaction with the aromatic pharmacophore of **19**, while being itself stabilized by a cation–π interaction with R228. Furthermore, the molecule's carboxylate group is anchored through a series of hydrogen bonds involving the backbone amides of E170 and H171 in the CCD, as well as an extended rotamer of K266 in the CTD. A particularly insightful discovery was the identification of a novel binding pocket at the CCD–CTD interface, created by a significant conformational shift of Trp131, which rotates approximately 120° upon CTD binding (Figure 16B). In the crystal structure, this pocket is occupied by a molecule of ethylene glycol that lies in direct van der Waals contact with the inhibitor. This finding has a high strategic value, as it suggests that future drug optimization could involve the elaboration of chemical moieties designed to exploit this specific void, potentially increasing both affinity and the genetic barrier to resistance. Parallel to these findings, further studies [133] have shed light on how resistance emerges; mutations like W131C and N222K act as “second‐shell” perturbations that alter the quaternary orientation of the CTD. This geometric shift destabilizes the drug‐induced polymer, allowing the virus to maintain functionality even in the presence of the inhibitor [133].

The specificity of these interactions was further explored by comparing the pyridine‐based ALLINI **21** and quinoline **19** [117]. While both derivatives induced higher‐order multimerization of full‐length WT IN tetramers, compound **19** showed a broader specificity, interacting with both dimers and tetramers in 2:2 ratio, whereas derivative **21** showed higher selectivity for tetramers [125]. Superimposition of the co‐crystal structures of compound **21** or **19** in complex with IN CCD aided to rationalize these observations. Similarly to **24** (Figure 11), compound **19** (PDB: 6NUJ) was found to localize deeper inside the V‐shaped pocket, allowing symmetrical CCD–**19**–CTD interactions between both IN dimers and tetramers. Differently, analysis of the X‐ray structure of derivative **21** (PDB: 4O55) revealed that the bulky benzimidazole group projects outside this pocket, preventing dimer–dimer symmetry while optimally positioning two IN tetramers for symmetrical CCD–inhibitor–CTD interactions.

Interestingly, while compounds proved to induce higher‐order multimerization of only full‐length IN, truncated protein constructs displayed substantial resistance to aggregation. This finding indicated that also the NTD of IN plays a role in compound‐induced higher‐order oligomerization. Indeed, although the domain does not bind the inhibitor directly, site‐directed mutagenesis of the NTD and the CCD–CTD linker compromised tetramer stability and conferred resistance to compound **21**‐induced oligomerization. This suggests that the NTD contributes to stabilizing the functional tetramer by interacting with the α‐helical linker connecting the CCD and CTD.

Finally, to explore a possible eutomer effect, single enantiomers of **21** were investigated. The enantiomer (−)−**21** displayed higher antiviral efficacy than (+)−**21** in MT‐4 cells ((−)−**21**: EC_50_ =6.28 nM); ((+)−**21**: EC_50_ = 203.9 nM) and demonstrated improved metabolic stability in rat and human microsomes compared with both racemic mixture and compound **24**. Notably, the eutomer (−)−**21** showed prominent antiviral efficacy against the DTG‐resistant mutant HIV‐1_NL4−3_ containing IN N155H/K156N/K211R/E212T substitutions (IC_50_ = 0.70 nM), highlighting the potential for ALLINIs to complement INSTI‐based HIV‐1 therapies [117].

Taken together, the growing understanding of ALLINI SARs and mechanism of action, greatly supported by structural insights, has unveiled their underlying basis of function, resulting in the design of increasingly potent and pharmacologically optimized derivatives. This progress has facilitated the transition from early lead compounds to optimized preclinical candidates, some of which have undergone clinical evaluation.

### 2.10 Emerging ALLINIs in Clinical Development

Over time, studies have clarified the structural and molecular basis of ALLINI function, identifying inhibitor‐induced IN multimerization as the key mechanism of action. However, how this process affected viral maturation and infectivity remained unclear until the discovery of a previously unrecognized, noncatalytic role of IN in HIV‐1 biology. Specifically, IN was shown to directly bind the viral RNA genome within virions, highlighting a fundamental role in viral morphogenesis [13, 135]. These findings provided unprecedented insights into the mode of action of ALLINIs, indicating that such inhibitors primarily act by inducing aberrant IN multimerization and disrupting IN–viral RNA interactions. This leads to the mislocalization of viral RNPs outside the capsid, occupying an eccentric position between the core and the viral membrane, in contrast to mature infectious virions where RNPs are enclosed within the capsid core [112]. Importantly, representative ALLINIs, such as **11** and **17** (Figure 6), were found to impair the binding of WT IN to viral RNA in virions [135]. Consequently, ALLINI‐treated virions display mislocalized RNP complexes and are noninfectious. Notably, nucleocapsid–RNA interactions remain unaffected, indicating that disruption of IN–RNA binding is a specific and critical determinant of the observed phenotype. Mechanistically, ALLINI‐induced multimerization likely sequesters key RNA‐binding residues within the IN CTD, thereby preventing productive interaction with the viral genome. These findings clarify the molecular basis of ALLINI activity and establish that their primary antiviral effect at the late stage of replication arises from the alteration of functional IN multimerization and the consequent disruption of IN–RNA interactions, thus resulting in defects in virion maturation [13, 112, 135, 136].

However, despite significant advances in unveiling the structural and functional basis of ALLINI efficacy, the search for effective in vivo antiretroviral agents has progressed more slowly. Indeed, important limitations in ALLINI efficacy have emerged, and the encouraging in vitro potency of these compounds has not always been translated into consistent in vivo outcomes. As a result, only a small subset of ALLINIs has progressed through preclinical development to reach the stage of clinical candidates. This discrepancy has been largely attributed to safety issues associated with experimental ALLINIs, whose development was halted due to toxicity observed in preclinical animal studies [137, 138, 139]. A representative example is the highly potent in vitro pyridine‐based ALLINI **21** (Figure 11) and its derivatives, for which no further clinical development has been reported, highlighting the challenges associated with translating potent ALLINI scaffolds into viable therapeutic candidates. Similar limitations have been observed across this class, where issues related to PK, toxicity, and overall in vivo performance have hindered clinical progression [117, 137, 138, 139].

### 2.11 Toward the Discovery of *In Vivo* Effective ALLINIs

**FIGURE 17:**
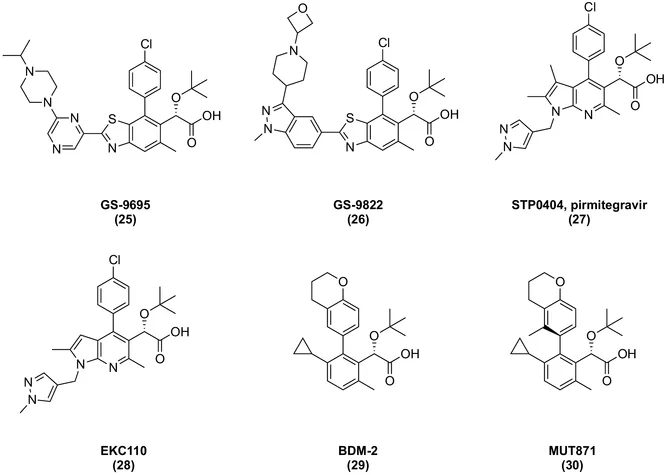
Chemical structures of ALLINIs **25**–**30**.

Next‐generation ALLINIs such as GS‐9695 and GS‐9822 [138] (**25** and **26**, Figure 17) were developed to significantly improve antiviral potency and the genetic barrier to resistance compared with first‐generation candidates like **19** (Figure 6) [138]. The compounds share a common 2‐substituted benzothiazole core incorporating the α‐(*tert*‐butoxy)acetic acid and *p*‐chlorophenyl moieties typical of most ALLINIs, while featuring alternative substituents in position 2 of the central core. Optimization of the initial lead **25** through introduction of a substituted indazolyl group at the 2‐position resulted in compound **26**, which exhibited significantly improved metabolic stability and an increased barrier to resistance. The compound proved to inhibit the IN–LEDGF/p75 protein–protein interaction, demonstrating a 13‐fold increase in potency over the thienopyridine **16** [137] which notably translated into exceptional antiviral efficacy in infected cells (Table 3). Beyond acute inhibition, **26** was found to induce a “block‐and‐lock” phenotype by retargeting residual integration away from methylation marks usually highly favored by HIV toward repressive heterochromatic regions, establishing a reservoir that is highly refractory to reactivation. However, despite its prominent profile, the clinical development of these rigid scaffolds was halted due to species‐specific urothelial toxicity observed in cynomolgus monkeys, characterized by transitional cell vacuolation and bullous formation [142]. This toxicity is rooted in a unique structure‐toxicity relationship governed by the ionization state of the molecules. Indeed, **26**, with pKa values of 4.2 (carboxylic acid) and 5.8 (piperidine/oxetane), and **25**, with pKa values of 4 and 7.8, exist as rigid zwitterions within the acidic urinary pH range of primates, including humans (pH 5.5–7.4). In contrast, the alkaline urine of rats (pH 7.3–8.5) prevents this specific ionization. Surface activity measurements of **26** revealed an exotic transition from an adsorbed monolayer to a bilayer at the air/water interface specifically at pH = 5; this transition, driven by strong intermolecular associations between zwitterionic molecules, is highly disruptive to urothelial membrane integrity. Notably, this “detergent‐like” behavior is absent in in the quinoline‐based ALLINI **19**, which lacks the piperidine/piperazine moieties and transitions directly from a neutral to a cationic species without entering a zwitterionic phase with decreasing pH [142].

**TABLE 3:** Biological activity of compounds **25**–**30**.

Compounds	**IC**_**50**_ **IN‐LEDGF binding,** **μM** a	**EC**_**50**_ **for aberrant IN multimerization,** **μM** b	**Antiviral efficacy (EC**_**50**_ **, μM)** c	**CC**_**50**_ **(MT‐4, μM)** d	References
**25**	ND	ND	0.0012 ± 0.0002 (MT‐4)	7.1 ± 1.4	[138]
**26**	0.07 ± 0.02	ND	0.0022 ± 0.0003 (MT‐4)	4.7570 ± 0.5972	[137]
**27**	0.190 ± 0.07	0.147 ± 0.02	0.00166 ± 0.00032^e^ (MT‐4)	>10	[139]
**28**	ND	ND	0.0045 ± 0.0002 (HEK293T)	ND	[140]
**29**	0.047 ± 0.014	0.020 ± 0.011^f^	0.0087 ± 0.0028 (MT‐4)	69 ± 8	[141]
**30**	0.014 ± 0.005	0.031 ± 0.020^f^	0.0031 ± 0.001 (MT‐4)	46 ± 11	[141]

Abbreviation: ND, not determined.

a Concentration required to inhibit the in vitro IN‐LEDGF/p75 interaction by 50%.

b Concentration required to induce IN multimerization of IN by 50%.

c Effective concentration required to reduce HIV‐1‐induced cytopathic effect by 50%; cell lines are indicated in brackets.

d Cytotoxic concentration reducing MT‐4 cell viability by 50%.

e Expressed as IC_50_.

f Expressed as activation concentration (AC_50_).

### 2.12 The Discovery and Development of Pirmitegravir

In the search for potent ALLINIs with robust efficacy and safety profile in in vivo models, the identification of STP0404 [139] (pirmitegravir, **27**, Figure 17) reported by ST Pharm (Seoul, South Korea) has marked a major milestone in the field [139].

Compound **27** retains the hallmark features of previously reported ALLINIs (i.e., a central scaffold with a 4‐substituted phenyl group along with a 2‐(*tert*‐butoxy)acetic acid moiety), while displaying a pyrrolopyridine core and an *N*‐methyl(pyrazolyl)methyl substituent as key structural points of divergence. The compound displayed excellent antiviral activity at low nanomolar concentrations with negligible cytotoxicity in human cells (Table 3). Due to its stereogenic center, the compound exists as two enantiomers; the *S*‐enantiomer displayed potency comparable to the racemate, while both outperformed the *R*‐enantiomer. Serial passage in the presence of the compound promoted the development of Y99H and A128T mutant strains, which exhibited cross‐resistance to the quinoline‐based ALLINI **19**. By contrast, compound **27** remained effective at nanomolar concentrations against strains with variations at the polymorphic positions 124 and 125.

**FIGURE 18:**
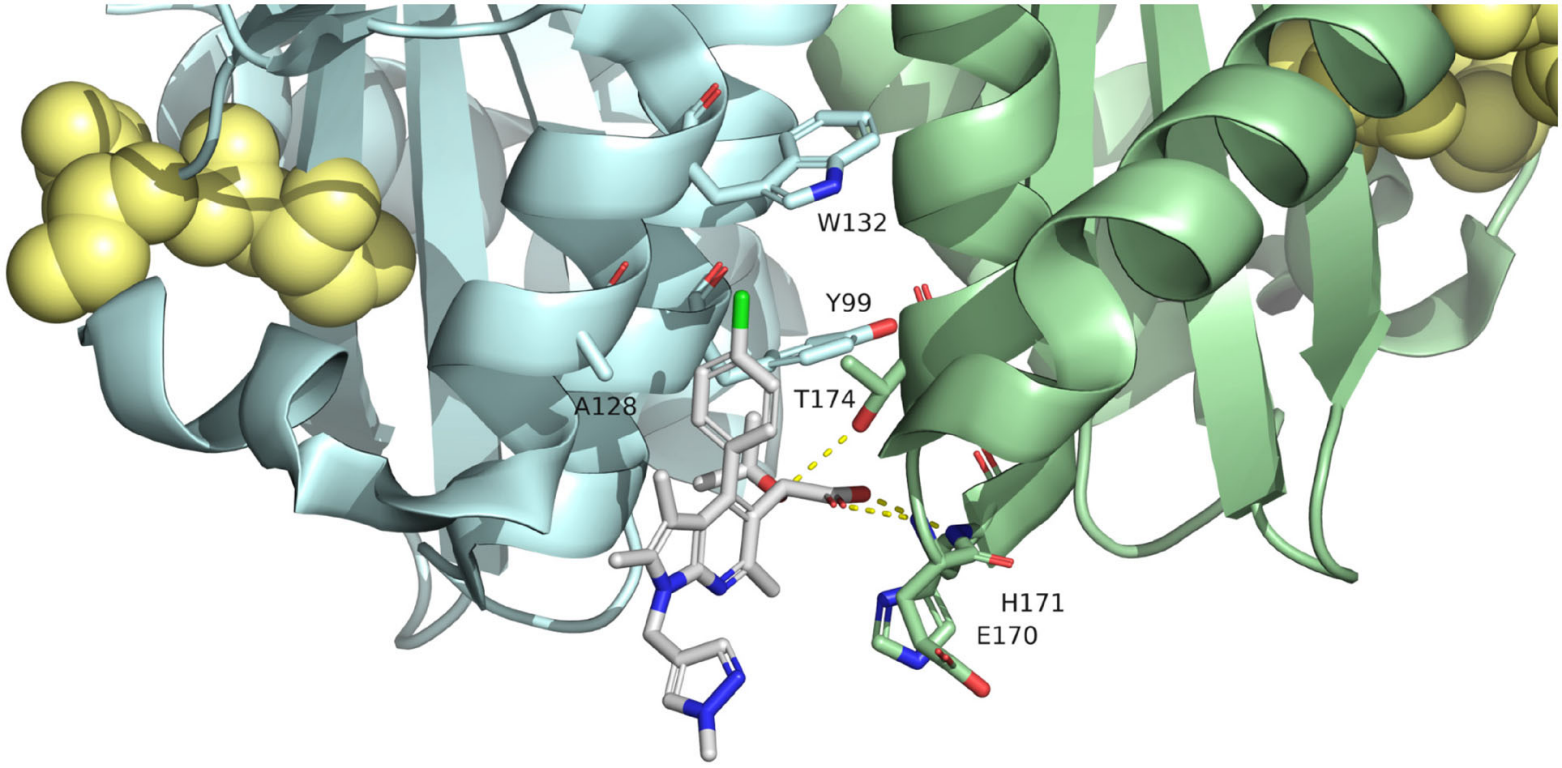
Crystal structure of compound **27** bound to HIV‐1 IN CCD dimer interface (PDB: 7KE0). CCD dimer chains are represented with cartoons and colored in light cyan and light green, respectively. Compound **27** is represented as white sticks, with nitrogen, oxygen, and chlorine atoms highlighted in blue, red, and green, respectively.

The X‐ray structure of HIV‐1 IN CCDF185H in complex with compound **27** (PDB: 7KE0) revealed that the inhibitor occupies the V‐shaped pocket formed by the two CCD subunits, establishing interactions comparable to those reported for other ALLINIs (Figure 18), while the pyrazole group projects outward from the core scaffold and does not directly contact the CCD dimer. Structural inspection further helped to explain the reduced activity of compound **27** against HIV‐1 variants carrying A128T and Y99H substitutions. The close proximity of residue A128 to the pyrrolopyridine moiety suggested that replacement with the bulkier, polar threonine may introduce steric constraints that hinder inhibitor binding. Likewise, Y99 lies deep within the V‐shaped pocket and plays a role in CCD–CCD contacts; mutations at this position are therefore likely to alter the pocket architecture and negatively affect ligand accommodation.

In addition, the compound proved to inhibit IN–RNA binding (IC_50_ = 0.020 µM) and viruses produced from cells treated with the inhibitor showed their viral RNA genomes outside of the capsid. Consistently with other ALLINIs, derivative **27** was found to induce higher‐order IN multimerization, disrupt IN–LEDGF binding, and primarily inhibit the late stages of viral replication. Importantly, the compound demonstrated favorable PK and safety profiles in preclinical models (rats and beagle dogs), including minimal interaction with drug‐metabolizing enzymes and good oral bioavailability, supporting its potential for oral once‐daily administration. The compound has demonstrated favorable safety and PK profiles in Phase 1 and nonclinical studies and is the first HIV‐1 ALLINI to enter a POC clinical trial. The interim analysis results of the first two completed cohorts (200 and 400 mg) highlighted promising results, while results of Cohort 3 (600 mg) are expected within 2026 [139, 143, 144].

**FIGURE 19:**
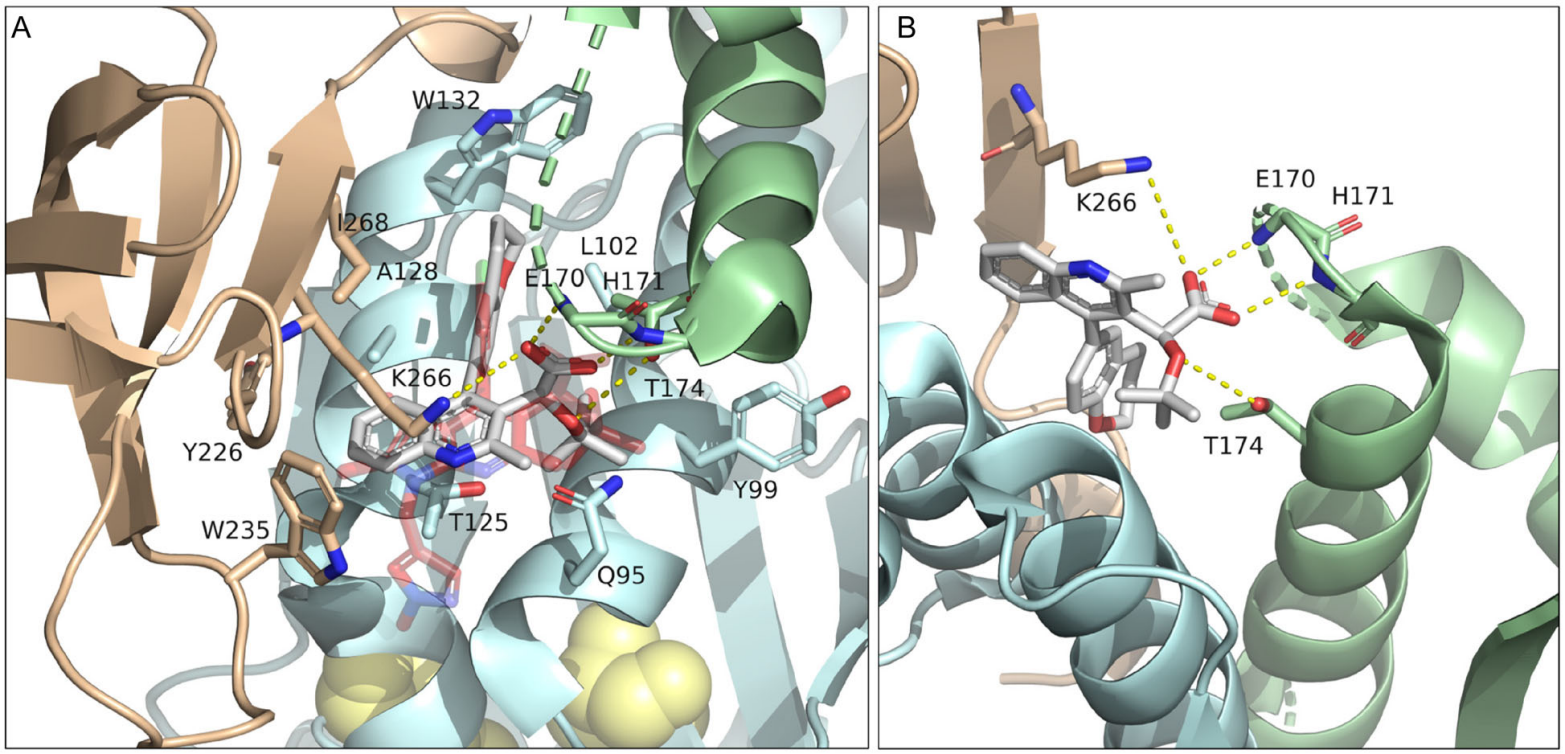
Crystal structure of compounds **17** and **27** bound to HIV‐1 IN CTD–CCD interface (PDBs: 8A1Q and 8A1P). (A) Cartoon and superimposed representation of compounds **17** and **27**. (B) Zoomed‐in, alternative perspective focusing exclusively on compound **17** to clearly illustrate its binding interactions. CCD dimer chains are colored in light cyan and light green, while the CTD is shown in gold. Compound **27** is depicted in red and transparency, while **17** is depicted as white sticks, with nitrogen, oxygen, and chlorine atoms highlighted in blue, red, and green, respectively.

To gain detailed structural insights into their mode of action, high‐resolution cocrystal structures of a CTD–CCD fusion construct with **27** and representative quinoline‐based ALLINI **17** were solved (PDBs: 8A1Q and 8A1P) (Figure 19) [145]. Both compounds localized at the CCD dimer interface, where their carboxylate groups formed bidentate hydrogen bonds with residues E170 and H171; in addition, the *tert*‐butoxy and bulky aromatic moieties were found to establish hydrophobic contacts with side chains from both CCD subunits (Q95, Y99, L102, T125, W132, T174, and M178). The main scaffolds of the compounds were found to protrude from the CCD dimer and engage in π–π stacking interactions with CTD residues Y226 and W235, promoting ionic interactions between K266, E170, and the inhibitor carboxylates. Additional interactions include a parallel π–π stacking between the methylpyrazole group of **27** with W235 and the hydrophobic contacts formed by the chromanyl group of **17** and the chlorophenyl substituent of **27** with the CTD residue I268 (Figure 19). By strengthening CTD–CCD contacts through the engagement of key conserved residues of HIV‐1 IN (E170, Y226, K266, W235, and I268), these compounds behave as authentic molecular glues [145].

Unlike classical molecular degraders, which stabilize interactions between E3 ubiquitin ligases and their target proteins to exploit the ubiquitin–proteasome system for target elimination, ALLINIs behave as nondegrading molecular glues [146]. As such, these inhibitors enhance the glue‐like stabilization of an otherwise transient or unfavorable interaction interface, thereby inducing the rapid, higher‐order multimerization and hyper‐aggregation of IN, which ultimately blocks IN functionality and affects viral maturation.

The growing interest of drug discovery campaigns in the search for nondegrading molecular glues, combined with the key structural insights recently described regarding the ALLINI‐induced glue‐like stabilization, will serve as key drivers for the rational design of next‐generation inhibitors.

**FIGURE 20:**
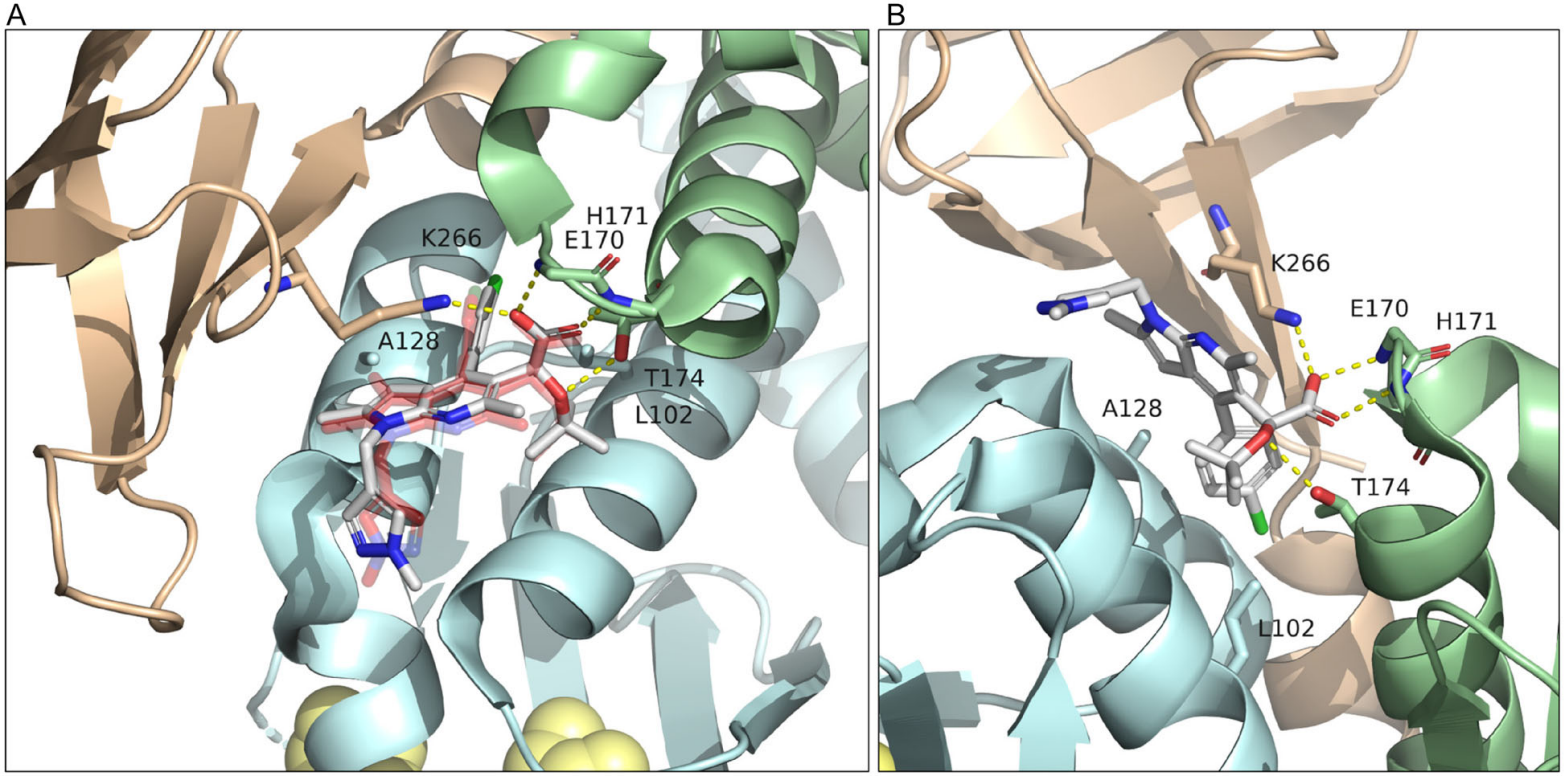
Crystal structure of compounds **27** and **28** bound to HIV‐1 IN CTD–CCD interface (PDBs: 8A1Q and 8T5B). (A) Cartoon and superimposed representation of compounds **27** and **28**. (B) Zoomed‐in, alternative perspective focusing exclusively on compound **28** to clearly illustrate its binding interactions. CCD dimer chains are colored in light cyan and light green, while the CTD is shown in gold. Compound **27** is depicted in red and with transparency, while **28** is depicted as white sticks, with nitrogen, oxygen, and chlorine atoms highlighted in blue, red, and green, respectively.

Further analyses of resistance‐associated mutations selected under compound pressure, particularly Y99H and A128T, revealed reduced viral infectivity and a marked decrease in susceptibility, especially in double mutants [140]. Although these substitutions had limited impact on direct inhibitor binding to the CCD, they significantly disrupted CTD recruitment to the CCD–inhibitor complex. Structural comparisons indicated that A128T introduces steric clashes with CTD residues (notably Y226 and I268) and alters local positioning through additional interactions, ultimately impairing CCD–CTD assembly. Moreover, the orientation of the 3‐methyl group toward residues A128/T128 may further restrict access of the inhibitor to the V‐shaped pocket, contributing to reduced efficacy. To enhance deeper positioning within the binding pocket, a desmethyl analog of compound **27**, referred to as EKC110 [140] (**28,** Figure 17), was synthesized. This derivative showed approximately 14‐fold higher potency against the HIV‐1(Y99H/A128T IN) mutant and effectively induced aberrant IN multimerization in both WT and mutant enzymes. Structural analyses of **28** in complex with WT and mutant CCD (PDB: 8S9Q), as well as CTD–CCD constructs (PDB: 8T5B), revealed that the absence of the 3‐methyl group allows **28** to shift its chlorobenzene moiety deeper into the CCD–CCD cavity toward L102 (Figure 20). This repositioning facilitates a unique anchoring of the CTD, where the inhibitor's carboxylate establishes a direct ionic interaction with K266, effectively bypassing the unfavorable T128‐T124 hydrogen bond network induced by the A128T mutation. Compared with **27**, compound **28** promotes a distinct and more stable CTD orientation that is resilient to resistance‐associated structural shifts. Overall, these findings clarify the structural basis for the improved inhibitory potential of **28**, which could serve as a robust alternative to **27** due to its superior genetic barrier to resistance. Furthermore, these insights provide deeper guidance for the rational design of new, nondegrading molecular glues endowed with enhanced efficacy and resistance barriers.

### 2.13 The Discovery and Development of BDM‐2

To further expand the chemical space for this class of inhibitors, a new series of ALLINIs with highly promising properties has been recently reported by Biodim (Romainville, France). While sharing some similarities with previously described inhibitors, these derivatives differ in that they lack the typical central heterocyclic core. Instead, they feature a phenylacetic scaffold bearing either a 2‐*tert‐*butoxy (e.g., BDM‐2, **29** [141], and its methyl analog MUT871, **30** [141], Figure 17) or a 2‐cyclopropyloxy group [141]. Additionally, they incorporate a cyclopropyl group on the central phenyl core while retaining the 4‐chromanyl moiety (or related substituents) found in previously described ALLINIs, such as compound **18**. Comparative studies including previously reported ALLINIs and such phenylacetic derivatives showed distinct activity profiles. While compound **30** was the most effective at disrupting the IN–LEDGF/p75 interaction, derivative **29** demonstrated the highest potency in promoting IN aggregation (Table 3). Overall, the compounds were more potent in inducing IN multimerization than inhibiting IN–LEDGF binding, which is consistent with the concept that ALLINIs primarily act as IN–IN molecular glues rather than simple disruptors of the IN–LEDGF interaction. Both derivatives **29** and **30** also demonstrated strong antiviral efficacy against clinical isolates, outperforming RAL in this panel. Consistent with previous findings, the inhibitors were more active in multiple‐round assays, with compound **30** being the most active (EC_50_ = 3.1 nM). While the reference ALLINI **19** exhibited a 27‐fold difference in EC_50_ values between single‐ and multiple‐round assays, the new derivatives showed significantly higher ratios, ranging from 100 to 200. Interestingly, a direct correlation between activity in single‐round infection assays and the ability to disrupt IN–LEDGF binding was observed. Accordingly, the most potent inhibitor of IN–LEDGF/p75 interaction **(30)** exhibited the strongest antiviral activity in the single‐round infection assay (EC_50_ = 0.63 µM); conversely, the weakest inhibitor of the series demonstrated the lowest antiviral effect in the single‐round infection assay (EC_50_ = 9.2 µM), resulting in a remarkable EC_50_ ratio of 767 between the two assay types.

**FIGURE 21:**
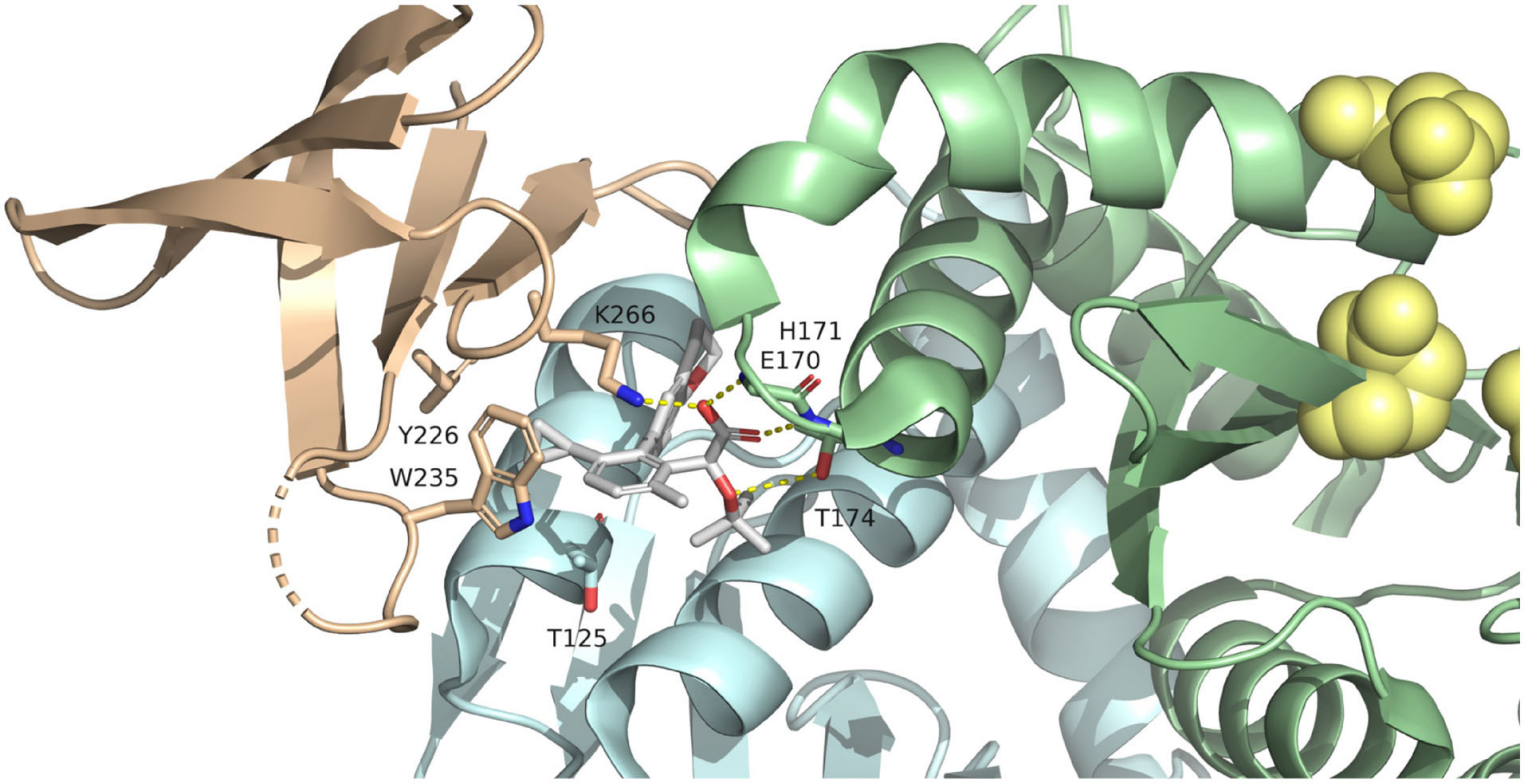
Crystal structure of compound **29** bound to HIV‐1 IN CTD–CCD interface (PDB: 8CBR). Chains are represented with cartoons. CCD dimers are colored in light cyan and light green, respectively, while the CTD is shown in gold. Compound **29** is depicted as white sticks, with oxygen atoms highlighted in red.

Notably, derivatives **29** and **30** retained their efficacy against primary isolates carrying polymorphisms at IN positions 124 and 125. Resistance mutations selected by compound **29** included T174I, Y99H, A128T, H171Q, and N222K, with T174I exerting the strongest negative impact on antiviral activity and viral fitness. Conversely, analogs of derivative **29** endowed with a α‐cyclopropyloxy group on the acetic moiety exhibited a lower EC_50_ fold change relative to WT HIV‐1, indicating that they possess a higher genetic barrier toward such mutation compared to **29**. Structural analysis of compound **29** in complex with previously described CTD–CCD construct (PDB: 8CBR) further clarifies these observations. The inhibitor is anchored by hydrogen bonds with the main‐chain amides of E170 and H171, while the T174 side chain establishes both hydrogen‐bonding and van der Waals interactions with the *tert*‐butoxy moiety of the inhibitor, explaining the loss of activity upon T174I substitution due to significant steric hindrance. Furthermore, this structure reveals how **29** acts as an efficient molecular glue by recruiting the CTD through a salt bridge between the inhibitor's carboxylate and K266. The compact benzene scaffold also allows W235 to approach the CCD more closely than in other ALLINI complexes, resulting in a distinct pivoting of the entire CTD domain that likely contributes to the unique multimerization profile of this series. In contrast, 2‐cyclopropyloxy analogs were less affected by the T174I mutation, likely owing to their more compact structure (Figure 21).

Importantly, the INSTI EVG retained full activity against ALLINI‐resistant mutants, and no antagonism was observed between compound **29** and approved antiretrovirals, while moderate to strong synergy was detected. Owing to its favorable profile, compound **29** advanced to clinical development: phase 1 studies in healthy volunteers reported good tolerability and PK properties, with no serious adverse events. Thus, together with derivative **27**, compound **29** currently represents one of the most advanced HIV‐1 ALLINIs under clinical investigation [141, 147].

## 3 Dual Inhibitors of the HIV‐1 IN Catalytic Site and IN−RNA Interactions

While the structural and molecular determinants of ALLINIs efficacy have been extensively characterized, the direct interaction between IN and viral genomic RNA represents the critical link in explaining how these inhibitors impair virion maturation. As previously discussed, this noncatalytic role of IN is indispensable for proper particle morphogenesis. The disruption of the IN–RNA interface by established ALLINIs, such as **11**, **17**, and **27** (Figures 6 and 17) leads to the formation of noninfectious eccentric particles, which confirms this interface as a strategic therapeutic target [135, 139].

Consistent with this mechanism, recent research has focused on the development of quinolinonyl derivatives as dual‐action inhibitors capable of targeting both the IN catalytic functions and the IN–RNA interaction [148]. This mechanistic study involved: (i) a series of bifunctional DKA quinolinone derivatives and their corresponding ester counterparts, featuring a *p*‐fluorobenzyl group or a hydrogen atom on the quinolinone nitrogen, and/or alkylamino groups at position 7 of the central core, some of which were previously described as inhibitors of IN catalytic functions [149]; (ii) a set of monofunctional DKA quinolinone derivatives; and (iii) a series of non‐DKA derivatives endowed with various arylmethyloxy groups at 6‐position of the central core, which have been previously described as RNase H inhibitors [150].

**FIGURE 22:**
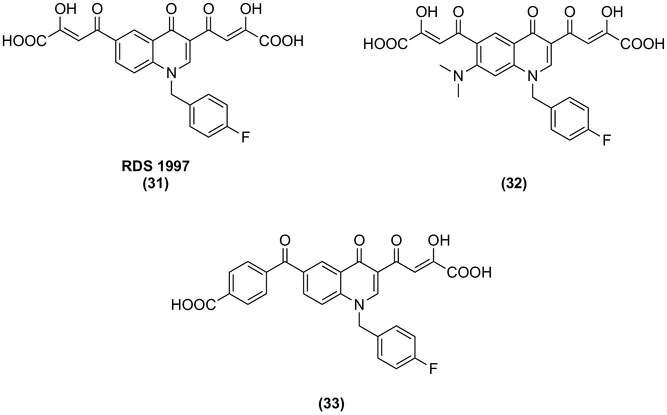
Chemical structures of inhibitors **31**–**33**.

Previous findings demonstrated that bifunctional DKA derivatives, exemplified by RDS 1997 [148, 149] (**31**, Figure 22) effectively inhibited both 3′‐P and ST reactions at nanomolar concentrations, while exhibiting low micromolar activity against HIV‐1 in acutely infected cells [149]. This feature notably differentiates this class of compounds from classical INSTIs, which typically display higher efficacy against ST over 3′‐P. Conversely, compounds endowed with a single DKA branch, including those bearing amino substituents at position 7 of the quinolone core, displayed marked selectivity for ST, showing up to two orders of magnitude higher selectivity over 3′‐P [149, 150, 151]. In addition, non‐DKA derivatives, obtained by shortening the DKA branch into a carboxylic acid function, displayed higher potency against RNase H compared to IN [150].

Most of the tested compounds inhibited the catalytic activity of IN in a LEDGF‐independent activity assay (Table 4). In general, DKA derivatives proved more active than non‐DKA analogs, with most of them showing potency within the submicromolar–nanomolar range, and derivative **31** displaying the highest potency (IC_50_ = 70 nM). SAR studies underscored the critical role of the *p*‐fluorobenzyl moiety for inhibitory potency, as its removal led to a 50‐fold reduction in potency. In addition, converting the DKA groups into ethyl diketoester moieties reduced activity by nearly 10 times (IC_50_ = 0.80 µM), indicating the relevance of the acidic portions to activity. In this series, the insertion of amino groups at position 7 of the central core proved a constructive strategy, and indeed 7‐amino substituted diketoester derivatives displayed potency within the submicromolar range. In particular, the insertion of a *N*,*N*‐dimethyl group at this position led to an IC_50_ of 480 nM, while the corresponding bifunctional DKA **32** (Figure 22) was nearly three times more active (IC_50_ = 181 nM). Interestingly, the monofunctional DKA derivative **33** (Figure 22) bearing a 4‐carboxybenzoyl group in place of the DKA branch at position 6 of the central core showed potency comparable to **31**. A similar trend was highlighted also in the ester series, with the ester analog of compound **31** showing similar potency to the ester congener of **33**, suggesting that the alternative acidic/ester moiety could mimic the second DKA/diketoester moiety of corresponding bifunctional derivatives.

**TABLE 4:** Biological activity of compounds **31**–**33**.

Compounds	**IC**_**50**_ **LEDGF‐independent** **IN catalytic activity,** **μM** a	**IC**_**50**_ **IN‐LEDGF binding,** **μM** b	**EC**_**50**_ **for aberrant IN multimerization,** **μM** c	**IC**_**50**_ **IN−RNA** **binding, μM** d	** Antiviral efficacy (EC**_**50**_ **, μM)** e	** CC**_**50**_ **,** **μM** f	References
**31**	0.070 ± 0.01	Q^g^	Q	0.84 ± 0.002	4.29 (H9)	>200 (U937)	[148, 149]
**32**	0.181 ± 0.06	AF^h^	Q	0.75 ± 0.06	ND	ND	[148]
**33**	0.0768 ± 0.011	Q	AF	1.57 ± 0.11	ND	ND	[148]

Abbreviation: ND, not determined.

a Concentration required to inhibit the in vitro LEDGF/p75‐independent catalytic activity of IN by 50%.

b Concentration required to inhibit the in vitro IN‐LEDGF/p75 interaction by 50%.

c Concentration required to induce IN multimerization of IN by 50%.

d Concentration required to inhibit the in vitro IN−RNA binding by 50%.

e Effective concentration required to reduce HIV‐1‐induced cytopathic effect by 50%; cell lines are indicated in brackets.

f Cytotoxic concentration reducing cell viability by 50%; cell lines are indicated in brackets.

g Fluorescence quenching.

h Auto fluorescence.

The compounds were also investigated for their ability to inhibit the IN–RNA binding (Table 4). Interestingly, bifunctional derivatives, including both DKA and ester analogs, displayed significant inhibitory potency, with IC_50_ values within the low‐micromolar‐submicromolar range. The most potent compounds were the bifunctional DKA derivatives **31** (IC_50_ = 840 nM), along with compound **32** (IC_50_ = 750 nM) and its ester analog (IC_50_ = 1.04 µM). Conversely, the removal of the *p*‐fluorobenzyl moiety from compound **31** led to a 1.5‐fold reduction in potency. A similar trend was highlighted in the ester series, indicating the halobenzyl group as a relevant feature for the inhibition of both IN–RNA binding and IN catalytic activity.

**FIGURE 23:**
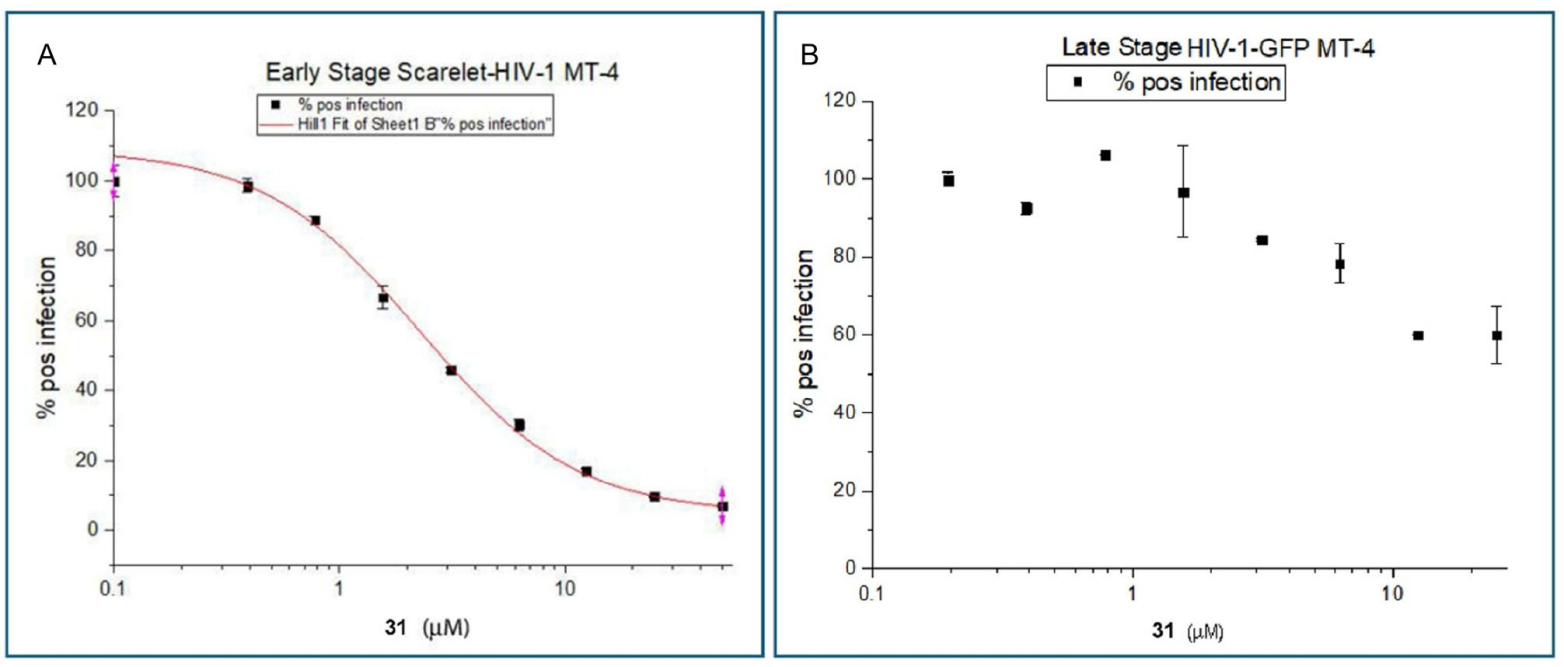
Antiviral activity of compound **31** in the early (A) and late (B) phases of HIV‐infection. Adapted with permission. [148] Copyright 2024, American Chemical Society.

The most promising inhibitors were assessed for their antiviral activity in both the early and late stages of HIV‐1 infection. While derivative **32** and its ester analog only partially inhibited the early steps of HIV‐1 infection without affecting the late steps of viral replication, this result remains consistent with their inhibitory potency toward IN catalytic functions. Differently, a more compelling profile was highlighted by derivative **31** (Figure 23). Indeed, the compound, previously reported to possess antiviral activity against HIV‐1 in acutely infected cells (EC_50_ = 4.29 μM) [149], displayed antiviral effects on both the early and late stages of HIV‐1infection, with an EC_50_ of 2.27 µM in the early stage and 40% inhibition at 25 µM in the late stage. Notably, the effects of **31** on both the early and late stages of HIV‐1 infection mirror its multitarget antiviral profile: its potent inhibition of both the 3′‐P and ST processes accounts for its early‐stage effect, while its interference with IN–RNA binding likely contributes to its late‐stage effect.

**FIGURE 24:**
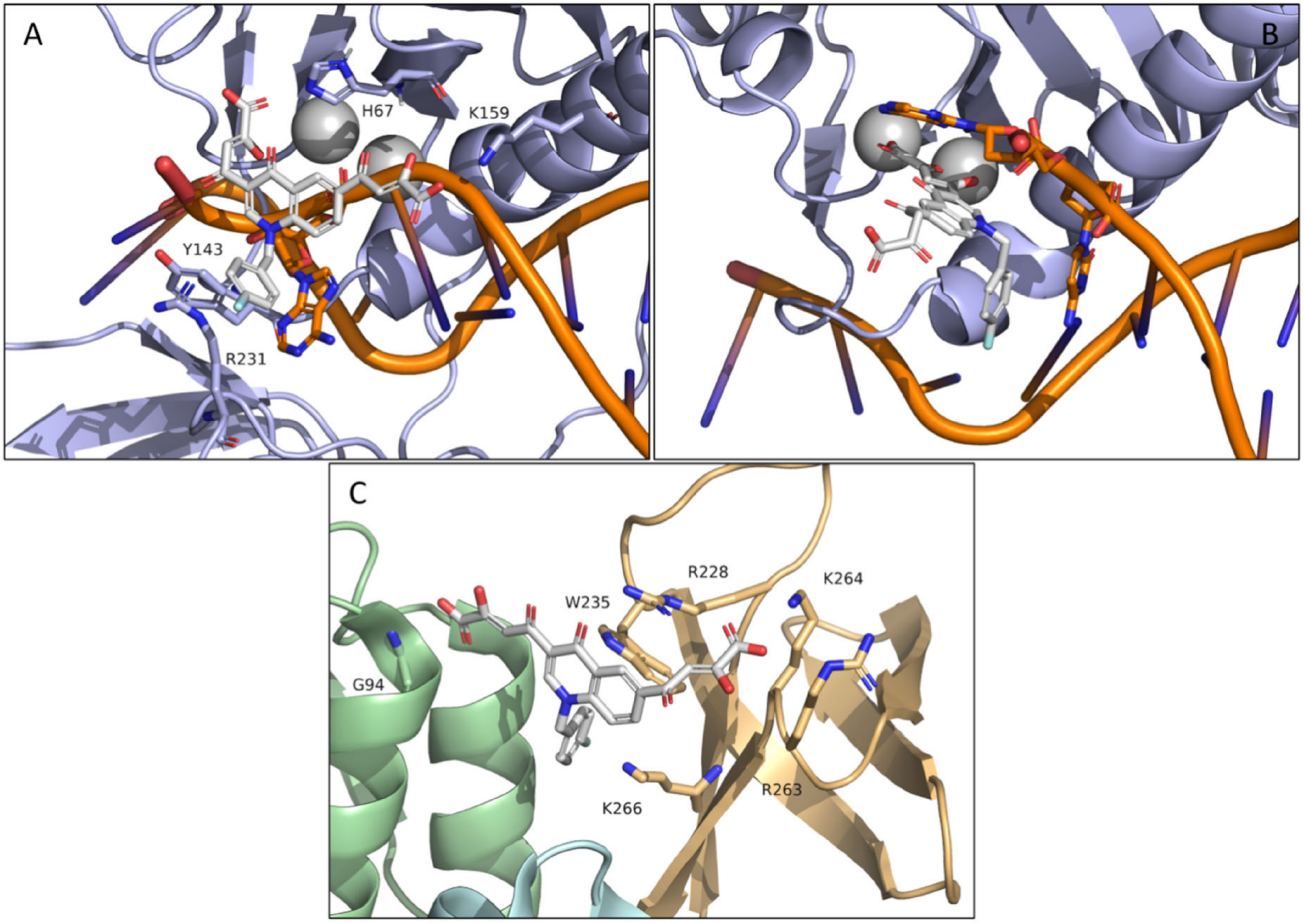
Proposed binding modes of **31**. Within the catalytic core, **31** inhibits (A) 3′‐P and (B) ST. In orange, the viral DNA. Magnesium ions are shown as gray spheres. (C) Within the CCD–CTD interface, **31** inhibits the IN−RNA binding.

The dual mode of action of **31** was further validated through computational studies [148] (Figure 24). Molecular docking simulations conducted on compound **31** provided a robust structural basis for understanding its multifunctionality as an inhibitor of both the HIV‐1 IN catalytic site and IN–RNA interactions. The study highlighted a clear distinction in structural requirements for catalytic inhibition, revealing that the presence of two DKA branches is essential for potent 3′‐P inhibition (Figure 24A). While one DKA chain chelates a single Mg^2+^ ion in the orthosteric site, the second branch serves as a critical anchor by forming a salt bridge with K159, which likely traps the ternary complex in a nonfunctional state. In contrast, ST inhibition requires only a single DKA chain to be effective (Figure 24B), as its oxygen atoms can successfully chelate both active‐site Mg^2+^ cations, explaining why monofunctional derivatives have been reported to only inhibit this specific step [152]. Beyond the catalytic sites, docking simulations suggest that **31** well occupies the CCD–CCD–CTD interface, where it interacts with key basic residues such as K264, R228, and K266 (Figure 24C). Since these residues are critical for RNA binding, this binding mode likely provides a structural explanation for the compound's ability to competitively disrupt interactions between IN and the genomic RNA. Indeed, two of the residues predicted to interact with the *p*‐fluorobenzyl group and one DKA branch of compound **31** (K264 and K266, respectively) were recently reported to affect the IN–RNA binding through mutagenesis studies [135]. The second DKA arm further stabilizes this allosteric binding via charge‐reinforced hydrogen bonding with the protein backbone.

Besides corroborating the mode of action of **31**, this study provides a valuable model for the search of IN inhibitors acting through an innovative mechanism of action. Moreover, based on structural insights from docking studies, chemical modifications to the compound's scaffold can be applied to allow the search for new derivatives endowed with enhanced potency, thereby paving the way for the development of structurally related analogs featuring this innovative mode of IN inhibition.

## 4 Summary and Outlook

As a hallmark of HIV‐1 replication, efforts to discover new anti‐HIV agents have focused extensively on the search for therapeutic agents targeting IN. These efforts have culminated in the development of first‐ and second‐generation INSTIs, which, owing to their remarkable antiviral efficacy and favorable PK profiles, have dramatically transformed ART regimens to become a cornerstone. However, the continuous selection of resistant variants remains a significant challenge, hindering the broad therapeutic utility of even these highly effective drugs. While resistance to second‐generation INSTIs has emerged clinically only recently, the decline in efficacy of first‐generation INSTIs occurred soon after their approval, limiting their long‐term use. These findings have prompted the exploration of alternative strategies to target IN through mechanisms of inhibition different from those of INSTIs.

Given the complex interplay between IN and human cofactors during integration, research has focused on targeting IN noncatalytically through the development of allosteric inhibitors capable of circumventing cross resistance issues and escape mutations within the catalytic site. In this context, ALLINIs have provided fundamental insights in the field, proving that targeting IN beyond its active site is a viable antiviral strategy. Indeed, ALLINIs have demonstrated efficacy against a broad range of viral clades, including those resistant to approved INSTIs. Furthermore, the fact that ALLINIs are not antagonistic to INSTIs, but rather demonstrate a likely synergistic or additive effect, suggests the great promise for their use in combination regimens. Targeting both the catalytic and allosteric functions of IN could likely delay the onset of resistance, indicating that INSTI‐ALLINI combinations could provide great therapeutic utility in the near future.

To establish ALLINIs as viable therapeutic options, extensive studies were conducted to optimize their drug‐like properties. This encompassed investigating not only their biochemical properties, but also their antiviral efficacy, genetic barrier to resistance, PKs, and toxicological properties. In this context, SAR studies, supported by biochemical and structural investigations, have been central to elucidating the key structural features impacting ALLINI activity. Exploration of chemical modifications has proven fundamental to understanding how specific characteristics impact the multimodal mode of action of these inhibitors.

While the 2‐(*tert*‐butoxy)‐2‐(4‐substituted aryl‐3‐yl)acetic acid moiety has been established as key pharmacophore of ALLINIs, fine‐tuning of structural features significantly impacts biological activity. This is specifically highlighted by compounds **27** and **29** (Figure 17), which, despite featuring alternative central scaffolds relative to the previously described ALLINIs, have shown prominent properties in both in vitro and in vivo models. Importantly, given their exceptional profiles, these ALLINIs represent a breakthrough in the field and are currently under investigations in clinical trials, further corroborating the great potential of ALLINIs as next‐generation therapeutics.

Research on ALLINIs has highlighted unexpected features in their mode of action. Although initial studies were primarily focused on developing protein–protein interaction inhibitors to disrupt the IN–LEDGF/p75 binding, the mechanism of action of ALLINIs has proven more complex. While disrupting protein–protein interaction could contribute to their efficacy, studies have found a major impact on IN multimerization. Notably, these investigations have been supported by recent advances in biophysical and structural biology, which have facilitated in‐depth investigations of the molecular basis of ALLINI function at near‐atomic detail.

Given their role in stabilizing CCD–CTD interactions to ultimately yield nonfunctional IN, ALLINIs have been recently categorized as molecular glues. However, differently from molecular glue degraders, which promote target degradation by recruiting E3 ligases, ALLINIs are more accurately described as nondegrading molecular glues [146]. Indeed, instead of exploiting the ubiquitin–proteasome system to eliminate disease‐associated proteins, ALLINIs work by stabilizing protein interfaces. This disrupts the structural and functional features of IN, inducing rapid, higher‐order multimerization and hyper‐aggregation of IN that ultimately impairs virion morphogenesis. Exploiting such mechanism is of great interest in modern drug discovery, with the development of nondegrading molecular glues becoming a major focus in the field. These findings, combined with the unprecedented structural insights from recent studies and extensive SAR data from past years, hold great promise for the rational design and optimization of next‐generation ALLINIs in the near future.

Furthermore, given their multimodal nature, ALLINIs have served as relevant investigational tools to probe the inherently complex structural and functional features of IN. A paradigmatic example of this is found in structural studies that characterized the enzyme's complex multimerization states [134]. By using these inhibitors to stabilize IN complexes, it was revealed that the enzyme possesses an intrinsic propensity to form not only linear aggregates but also complex branched polymers with fractal‐like properties. These studies demonstrated that homomeric interactions between the CTD act as critical branching points that transform the enzyme into an inactive, gel‐like state. Without the stabilizing effect of ALLINIs as research tools, the full extent of this structural plasticity and the CTD's role in driving higher‐order multimerization might have remained hidden.

This deep understanding of the viral structural network highlights potential avenues for future drug design targeting specific atomic vulnerabilities. A particularly strategic prospect lies in the identification of a novel binding pocket at the CCD–CTD interface, created by a significant conformational shift of W131 [116]. This pocket, found to be occupied by an ethylene glycol molecule in crystal structures, offers a strategic opportunity to modify traditional scaffolds. Such refinements could increase binding affinity and raise the genetic barrier against common resistance mutations like T125 and A128, which disrupt the complex interplay between IN, the capsid, and the inhibitor.

In addition to their therapeutic potential, ALLINIs have served as fundamental tools for unveiling previously unrecognized aspects of HIV‐1 biology. The discovery that ALLINIs primarily affect the late stages of the HIV‐1 life cycle has aided in unraveling their major effect on maturation of viral particles through the inhibition of IN–RNA interaction. This feature, which was previously unknown in the field, has allowed researchers to gain additional insights into mechanisms underlying ALLINI function, revealing that the binding of IN to RNA is fundamental for virion infectivity. These structural insights provided the conceptual foundation for the latest breakthroughs in high‐resolution cryo‐EM [153]. Consequently, it is now clear that the ability of IN to multimerize is not merely a drug‐induced artifact but a critical native function. In mature virions, IN forms ordered filaments that serve as a structural scaffold to organize and anchor the viral RNA genome. The fundamental insights provided by ALLINI‐based studies allowed researchers to visualize IN's polymeric potential, enabling cryo‐EM to eventually confirm that this architecture is fundamental for proper virion morphogenesis and genome packaging. Overall, these impactful studies have provided key benchmarks for innovative, structure‐based drug discovery campaigns focused on unprecedented mechanisms.

Looking forward, the search for innovative mechanisms has led to the development of dual‐action inhibitors [148] capable of targeting both the IN catalytic site and the IN–RNA interface, offering a bifunctional model that could drastically limit viral escape routes.

The future of HIV‐1 therapy lies in this continued integration of medicinal chemistry and structural biology, turning every new insight into IN multimerization and IN–RNA binding interaction into a more effective therapeutic weapon. Ultimately, the true legacy of ALLINIs lies in their ability to bridge the gap between molecular inhibition and structural orchestration, turning the virus's own complexity into its ultimate vulnerability and providing the blueprint for a future where HIV‐1 is systematically outsmarted.

## Funding

This work was supported by EU funding within the Next Generation EU‐MUR PNRR Extended Partnership initiative on Emerging Infectious Diseases (Project no. PE00000007, INF‐ACT, Spoke 5), Next Generation EU‐MUR PRIN 2022 project code 20228NPP2Y, and Ateneo 2024 (project code RG124190BB0EB5C9).

## Conflicts of Interest

The authors declare no conflicts of interest.

## Data Availability

The data that support the findings of this study are available from the corresponding author upon reasonable request.
